# The Health and Development of Young Children Who Witnessed Their Parent’s Arrest Prior to Parental Jail Incarceration

**DOI:** 10.3390/ijerph18094512

**Published:** 2021-04-23

**Authors:** Julie Poehlmann-Tynan, Luke Muentner, Kaitlyn Pritzl, Hilary Cuthrell, Lauren A. Hindt, Laurel Davis, Rebecca Shlafer

**Affiliations:** 1 School of Human Ecology, University of Wisconsin-Madison, Madison, WI 53706, USA; muentner@wisc.edu (L.M.); kepritzl@wisc.edu (K.P.); hcuthrell@bop.gov (H.C.); 2 Department of Psychology, Loyola University Chicago, Chicago, IL 60660, USA; lhindt@luc.edu; 3 Department of Pediatrics, University of Minnesota, Minneapolis, MN 55455, USA; davis978@umn.edu (L.D.); shlaf002@umn.edu (R.S.)

**Keywords:** arrest, child, criminal justice system, delay, health, incarcerated parents, jail

## Abstract

Most U.S. incarceration occurs in jails, with more than 10 million annual admissions, and most individuals in jail are parents of minor children. In this short-term longitudinal study, we examined the health and development of young children who did or did not witness their parent’s arrest prior to parental jail incarceration. 228 individuals in 76 triads (incarcerated parents, children, at-home caregivers) were enrolled from four jails in two states. Jailed parents and caregivers reported on whether the child witnessed the parent’s arrest or crime. Children’s caregivers completed questionnaires about children’s emotional symptoms during the prior 6 months and demographics, as well as children’s emotional reactions to separation from the parent and child health at the initial assessment and 2 weeks later. Trained researchers conducted a developmental assessment with children while waiting to visit parents. Results of regression-based moderated mediation analyses indicated that when their emotional symptoms were high, children who witnessed parental arrest were more likely to have poorer health initially and more intense negative reactions to the parent leaving for jail. In addition, when children’s general emotional symptoms were low, children who witnessed their parent’s arrest were more likely to exhibit developmental delays, especially in their early academic skills, compared to children who did not witness the arrest. Witnessing the parent’s crime related to missed milestones in social and adaptive development. Findings have implications for policies regarding safeguarding children during parental arrest and referrals for health- and development-promotion services following parental criminal justice system involvement.

## 1. Introduction

### 1.1. The Health and Development of Young Children Who Have Witnessed Their Parent’s Arrest Prior to Parental Jail Incarceration

**Figure 1:**
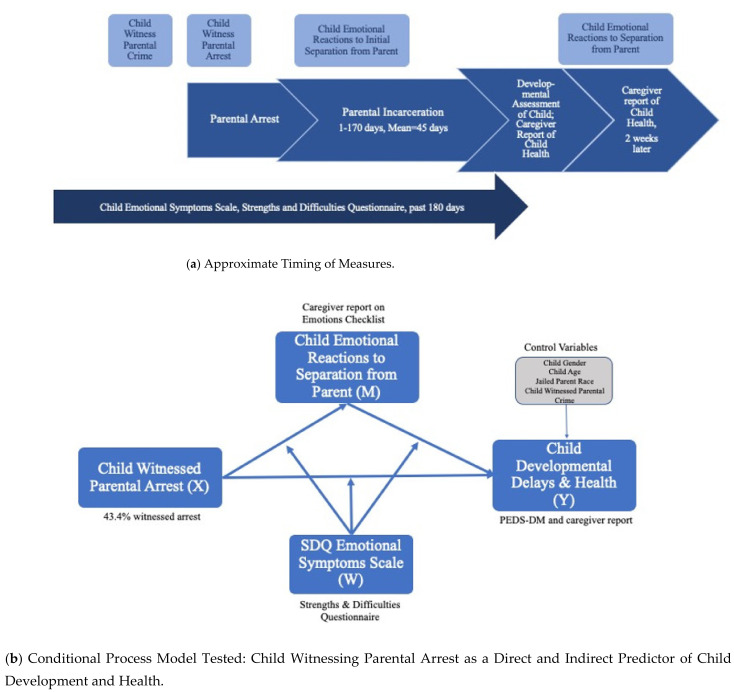
Study Overview.

U.S. children born in the last two decades are more likely to experience a parent being arrested and leaving for jail or prison than at any prior time in history, especially children of color and poor children [1]. Most incarceration in the U.S. occurs in jails, which are locally administered corrections facilities where individuals are held while awaiting conviction or sentencing or serving short-term sentences for misdemeanor crimes [2], with more than 10 million admissions annually [3]. Because about 65% of people in jail have minor children [4], millions of children are affected by parental jail incarceration [5]. Prior research has found that children with incarcerated parents are at elevated risk for experiencing behavior problems, mental health concerns, and academic challenges compared to their peers who have never had a parent in jail or prison, adjusting for other risks [5,6,7]. Criminologists and sociologists have argued that children and families should be studied across the entire spectrum of parental criminal justice involvement, including children’s experience of parental criminal behavior and arrest, not just incarceration [8], and that conditional processes linking parental criminal justice involvement with child outcomes receive more attention [7]. In this study, we examine processes that occur just before and during parental incarceration in jail (Figure 1a). Specifically, we examine whether witnessing a parent’s arrest, which may heighten children’s feelings of loss and stress, is associated with less optimal health and development in young children with jailed parents.

### 1.2. Parental Arrest and Incarceration and Feelings of Loss in Children

Scholars who study families involved in the criminal justice system have conceptualized parental incarceration as a type of ambiguous loss [9,10] because it involves the physical loss of a parent, often with little known about the details surrounding the separation--yet the incarcerated parent remains psychologically present to the family [11,12]. Ambiguous loss differs from typical loss or grief because it is unresolved, confusing, and highly stressful, in part because information that would allow feelings of finality or integration of the loss into one’s life is lacking [13,14]. As such, ambiguous loss and its accompanying stress can inhibit coping, problem solving, and decision-making in adults and children [13,14]. Contributing to the stress of the situation, families with a loved one in jail often experience material hardship and loss of resources because of the incarceration [15], in addition to stigma, which may prevent families from communicating clarifying details, such as reassuring the child that the parent is in a safe place and will be coming home on a certain day or time [9]. Jail incarceration may be particularly confusing because it often involves relatively short stays, the length of the stay is often uncertain, and parents may go in and out of jail, rather than serving a longer-term sentence [2,16].

When a young child is present during their parent’s arrest, the experience may add stress and confusion that intensifies the experience of ambiguous loss, including elevated distress and multiple or mixed emotions after the parent leaves [10,17], thus resulting in more challenges to health and development during or following parental incarceration. Indeed, Kampfner [18] reported that children who had witnessed their parent’s arrest remembered the experience vividly, even years later. Dallaire and Wilson [19] found that children with incarcerated parents (CIP) who witnessed their parent’s arrest, crime, or sentencing were more likely to have lower one-word receptive vocabulary, more symptoms of anxiety and depression, and lower self-regulation months after the event occurred compared to CIP who did not witness such events. Exposure to fathers’ arrests has been found to be associated with elevated physiological distress, except in the case of those children who see the high behavioral stress symptoms who actually see a blunted glucocorticoid response—trends mirrored in studies of PTSD [20]. The potential trauma of witnessing the parent’s arrest, combined with the experience of loss related to losing a parent to incarceration—especially when the parent is engaged or co-resident--could increase children’s stress levels and disrupt their development in multiple domains, especially emotional development, social development, health, and early learning. Such effects can be evident fairly quickly, in addition to having lifelong implications [21]. For example, Sharkey [22] found that homicides in Chicago neighborhoods were associated with lower vocabulary and reading skills in 5 to 17-year-old children who lived near the murder, with changes seen within a week of when the violence occurred.

### 1.3. Research on Witnessing a Parent’s Arrest

Although national statistics regarding the prevalence of children witnessing their parent’s arrest are not available, studies focusing on CIP or those in the child welfare system indicate that children are often present during parental arrest [23]. Depending on the method of data collection and children’s age, estimates indicate that between 22% and 41% of CIP have witnessed their parent’s arrest [19,23]. In an analysis of data from a national study of children age 8 and older involved in the child protective system, Phillips and Zhao [23] found that 38.7% witnessed the arrest of a household member; children self-reported the information as part of a violence exposure assessment, based on their responses to cartoon-like pictures depicting different kinds of violence. In other studies, incarcerated parents, children’s at-home caregivers, or both have reported on whether or not their children have witnessed the parent’s arrest [19]. Dallaire and Wilson [19] indicated that 26% of CIP between the ages of 7–17 had witnessed their parent’s arrest. However, other than Poehlmann-Tynan, Burnson, Runion, & Weymouth’s [24] and Muentner, Kapoor, Weymouth, Poehlmann-Tynan’s [20] study, the experiences of young children who have been present for their parents’ arrest has not been well-documented.

Because seeing and hearing a parent being arrested can be traumatic, especially when it is unexpected, sudden, or violent, there have been efforts to protect children from exposure to the arrest of a parent or caregiver [25]. For example, the International Association of Chiefs of Police (IACP) worked with the federal government and community groups to develop protocols that provide guidance on best practices for law enforcement when making an arrest when a child is present [26]. Although the training and protocols have been implemented at pilot sites and free webinars are offered by the federal government, many jurisdictions have not yet implemented the guidelines in a systematic way. There are notable exceptions, however. For instance, since 2007, the San Francisco Police Department has required officers to ask if a child was present, make arrests out of children’s view if possible, and attempt to find an adult relative who could care for children during or following the arrest [27]. More recently, the New York City Council passed a law requiring the New York Police Department to develop protocols that are sensitive to children during the arrest of a caregiver [28]. Such protocols indicate the need to train law enforcement officers regarding how to make the arrest of a parent less upsetting, fear-inducing, and traumatic for children. The IACP Model Policy includes determining whether children are present at the time of the arrest; calming the parent and supporting the parent in calming the child; having parents step out of the home or away from children so that children are not in view or earshot; avoiding using handcuffs in the presence of the child; explaining what will happen to the child and answering the child’s questions calmly and truthfully; and making sure that children are not alone during and following the arrest, preferably by ensuring that children are with a person known to them and that parents are given an opportunity to be involved in placement decision-making [26].

In the absence of such child-sensitive arrest protocols, parents are often handcuffed in the presence of children, and children typically react to their parent’s arrest with intense negative emotions, including general distress along with fear, confusion, anger, anxiety, and traumatic stress symptoms [19,24,29]. For example, Philips and Zhao [23] reported that witnessing the arrest of a household member (usually a parent) was associated with elevated post-traumatic stress symptoms in children who had been the subject of a child protective report. Qualitative findings indicate that some parental arrests witnessed by CIP include the parent being handcuffed at home, in the same room as the child or in a different room; outside of the home or in another location; or in a car with children inside or outside of it [24]. Sometimes children witness violence during the arrest, although this does not appear to be the norm [24,29,30]. Sometimes children are allowed to say good-bye to the parent, whereas in other cases children are not; additional differences include whether children are left at home with another parent, picked up from the arrest site by a relative, or transported to foster care in a police car or social worker’s vehicle [24,29]. Recent evidence has also pointed to the ways in which these experiences “get under the skin” of children, finding significant concern for physiological stress after witnessing fathers’ arrests [20]. Coping with witnessing parental arrest can be challenging for children and contribute to the ambiguity and stress surrounding parental loss, especially without implementation of child-sensitive police protocols.

Studying this sort of exposure to law enforcement and the criminal justice system during childhood is important given the connections between (vicarious) police contact on older children’s adjustment. Defined as witnessing a police stop or knowing someone who had law enforcement exposure, this vicarious contact has been found to be associated with lower educational achievement among urban teenagers [31]. Beyond this, it also has consequences for one’s psychological well-being, citing impaired mental health [32,33] and socio-psychological and behavioral outcomes [34] at rates that may disproportionately affect children dependent upon their race and gender [35].

In the present study, we examine incarcerated parents’ and at-home caregivers’ reports of whether or not children witnessed parental arrest and how much distress the child exhibited at the time in jurisdictions that did not have child-sensitive protocols in place. We explore how witnessing the arrest may interact with children’s existing emotional concerns in relation to: (1) children’s negative emotional reactions about the parent’s departure for jail and (2) children’ subsequent development and health.

The counterfactual to our argument regarding witnessing parental arrest has to do with parental supervision of children; specifically, whether or not children who witness the parent’s arrest are less likely to be supervised by parents and thus, be more likely to witness other negative or traumatic events, which may also affect their health and development. Because children witnessing parental crime is likely to be more under the parent’s control compared to witnessing the parent’s arrest, which may occur unexpectedly or suddenly, we explored: (a) whether or not witnessing the parent’s crime diminished the impact of witnessing parental arrest on children’s negative emotional reactions about the parent’s departure for jail and children’s subsequent health and development, and (b) whether witnessing the parent’s crime or arrest were interchangeable variables in our models.

### 1.4. Emotional Concerns, Health, and Development in Children with Incarcerated Parents

Behavior problems, emotional well-being, and academic success are among the most-studied outcomes in research focusing on CIP [36]. Studies have consistently found that children who have ever experienced the incarceration of a parent—especially of a father—are at risk for developing elevated externalizing behaviors across childhood and problems in elementary and middle school compared to children with parents who have never been incarcerated, adjusting for observed selection factors [7]. Some cognitive, language, and academic problems have been documented as well (e.g., [37]). For example, Haskins [38] found that paternal incarceration was associated with lower attention scores in boys and lower math problem solving scores in girls as compared to their same gender peers. Other studies have found elevated internalizing symptoms and emotional concerns among CIP as well, depending in part on the age of the child [39]. Despite parents perceiving their incarceration negatively influencing their children’s health [40], this consequence of parental imprisonment tends to receive less attention despite some health effects being documented in young children (e.g., [41,42]. Pointedly, younger children see increased odds of adverse sleep and eating behaviors as a result of their parents’ incarceration [43], elevated physiological distress [20], and ultimately face higher risk for psychopathology [44]. Studies focusing on adolescents [45] and adult retrospective reports of adverse childhood experiences have found links between childhood experiences of a household member’s incarceration and poor physical and mental health outcomes, including poor health-related quality of life [46], health behaviors [47], diabetes [48], and elevated depressive symptoms [49].

Other work that documents risk to children’s health while parents are incarcerated also call for the need to explore such associations in the context of cumulative disadvantage. In other words, it may be necessary to study incarceration in context as it contributes to co-occurring stressors and adversities. For instance, McCauley [50] finds an association toward heightened risky sexual health behaviors for young adults who experience household member incarceration but also suggest that household functioning, social inequality, and historical racism may be contributing factors. Similarly, Jackson and colleagues [51] discuss the additive role of childhood adversity, such as family death or divorce, when determining the magnitude of the role of parental incarceration on child health. These findings around the clustering of stressful life events for children in justice-involved families are not solely concentrated among young adults or even children, rather they carry significant weight in influencing maternal and infant health, as well [52]. In general, more work is needed to explore how stressful events, such as exposure to parental arrest, may be stress-related driver of detriments to child health.

In this study, we examined young children’s caregiver-reported health and observed developmental milestones in the areas of language, social and adaptive skills, motor development, and early literacy and numeracy. We also assessed children’s ongoing emotional symptoms (i.e., the tendency to be worried, withdrawn, anxious, or depressed), which may relate to children’s reactions to witnessing the parent’s arrest or to the current incarceration, or may reflect a child’s disposition or longer-standing problems.

Scholars argue that taking a developmental approach is particularly important when studying CIP, because reactions to and understanding of parental arrest and incarceration may change dramatically across early childhood, middle childhood, and adolescence [36]. Taking a developmental perspective means that one attends to children’s age and developmental competence in multiple domains. In research that has taken a developmental approach, young children are found to react to separation from their incarcerated parents with a variety of emotions and behaviors, including developmental regression, sadness, crying, confusion, anger, and worry [53]. Compared to older children, preschool age children are also less likely to understand what is happening when a parent is arrested or incarcerated because of relatively limited cognitive and language capacities (e.g., [36,53]), thus creating a high likelihood of experiencing stress related to ambiguous loss in young children. Taking a developmental approach also means examining child development over time. In this short-term longitudinal study, we were able to assess children’s health and emotional reactions to the parent’s departure for jail at the time of the initial assessment and 2 weeks later.

In addition to taking a developmental perspective, it is particularly important to study CIP in early childhood, especially given the high prevalence of parental incarceration among young children. Data from the National Study of Children’s Health indicate that among 3- to 8-year-old-children, rates of parental incarceration range from 5.1 to 6.7 percent, with cumulative rates peaking by the time children turn 9 years of age [5,54]. Moreover, young CIP appear particularly vulnerable to adverse childhood experience exposures compared to older CIP [55], and the negative short- and long-term developmental effects of intense or chronic stress in early childhood are well-documented (e.g., [21]). Finally, investigators analyzing at least three population-based datasets have found age-graded effects of parental incarceration on children’s well-being, with more effects when the incarceration occurred earlier in the child’s life [56,57,58]. 

### 1.5. Conditional Processes and Parental Incarceration

Only a few population-based studies in the existing CIP literature have explored possible mediators or moderators of the relation between parental incarceration and child physical health, mental health, behavior problems, or educational outcomes [39]). Within-group studies with targeted sampling can help fill this gap by examining heterogeneity in child development, health, and family processes, with the results possibly indicating the need for further study of factors rarely included in population-based parental incarceration studies (e.g., witnessing parental arrest or the child’s reaction to the parent leaving for incarceration; incarceration-related details). Moreover, within-group studies do not have the same problems with selection bias that are found in comparison group studies, and they allow in-depth exploration of heterogeneity in children’s experiences of parental incarceration. Of course, there are limitations as well (see Limitations section).

In this study, we test a conditional process model examining the association between witnessing a parent’s arrest and child health and development among 3- to 8-year-old children with parents in jailed. After presenting descriptive statistics, we test a model linking children’s witnessing of their parent’s arrest with children’s emotional reactions to separation from the parent cause by the parent’s incarceration, as moderated by children’s general emotional symptoms. We then extend the model to children’s developmental and health outcomes. By including whether or not the child witnessed the parent’s crime in a second set of models [19], we also examine the possibility that the findings may have resulted from exposure to other stressful and potentially traumatic events (i.e., witnessing the parent’s crime) rather than being specific to witnessing parental arrest.

Given that some studies have found particularly strong effects of parental incarceration on boys’ externalizing behavior, as well as differences based on child age (e.g., [7,59]), we include child age and gender in our models. In addition, we explore differences based on incarcerated parent gender and education, along with family income because children with incarcerated mothers experience more environmental risks, on average, than children with incarcerated fathers [60]. Because Black men and women are stopped and arrested more frequently and, on average, exposed to more violent police procedures than White men and women (e.g., [61]), we also examine parental race as a covariate.

### 1.6. Research Questions

Research questions focused on families in which the incarcerated parent lived with the child or was engaged in the child’s life prior to incarceration and included: (1) What proportion of young children witnessed the arrest or crime of a parent who was then incarcerated in jail, and did this differ by parental race? (2) What types of emotional reactions did caregivers report in young children following separation of the child from the parent because of parent’s incarceration, and did this change over time? (3) Did witnessing the parent’s arrest relate to children’s emotional reactions after the parent left for jail, conditional upon the child’s general emotional symptoms? We hypothesize that witnessing the arrest, combined with existing emotional symptoms, would relate to more negative emotional reactions following the parent’s incarceration. (4) Did witnessing the parent’s arrest relate to subsequent problems in child health or developmental delays (language, social, academic, or motor skills), conditional upon the child’s general emotional symptoms? We hypothesize that witnessing the parent’s arrest, combined with general emotional symptoms, would relate to more negative emotional reactions to the parent’s departure and less optimal child health and development. (5) Did emotional reactions to the parent’s departure mediate the relation between witnessing the parent’s arrest and child outcomes? We hypothesize that negative emotional reactions to the parent’s departure (an indicator of ambiguous loss) would partially mediate the relation between witnessing the parent’s arrest and child outcomes. (6) Do the above relations change when adding witnessing parental crime to the models? We hypothesize that witnessing parental arrest will relate to child outcomes, even controlling for witnessing the parent’s crime.

## 2. Materials and Methods

### 2.1. Sample

The sample included data from 228 individuals nested within 76 triads of children (aged 3–8 years, *M* = 5.5, *SD* = 1.8), their at-home caregivers, and their jailed parents who had lived with the child or had been engaged in their care prior to the incarceration. The participants were part of a larger multisite longitudinal intervention study focusing on incarcerated parents and their children [39]. In this analytic sample, there were nine incarcerated mothers and 67 incarcerated fathers, and 76 focal children, 53% of whom were boys. Children’s race/ethnicity included: 42% White, 29% Black/African American, 18% biracial or multiracial, 8% Latinx, and 3% Native American. Fifty-five (72%) caregivers were children’s mothers or stepmothers, 18 (24%) were grandparents, two (3%) were other relatives, and one (1%**)** was the child’s father. Caregivers ranged in age from 18 to 71 years, with a mean of 36 (*SD* = 13.2). The majority of caregivers (77.5%) had completed high school or a higher degree, and 51% were employed outside of the home. Caregivers’ monthly income ranged from $0 to $6000 (*M* = $1382, *SD* = $1199), and 76% reported that they received public assistance. Jailed parents had been incarcerated for less than 180 days (*M* = 46.3, *SD* = 41.4). Jailed parents’ demographic characteristics are presented in Table 1.

### 2.2. Recruitment Sources

Children with jailed parents were enrolled as part of a larger intervention study examining children’s experiences visiting their parents in jail. The original multisite randomized efficacy study randomized set out to evaluate new materials that Sesame Street had recently developed specific to young children who had incarcerated parents, which included videos, a storybook, and a guide and tip sheet for caregivers aimed at supporting families while parents were away. Participating incarcerated parents and their children into either treatment or control groups; those families in the treatment group were given access to the Sesame Street materials and the children watched a newly developed episode of the show where a Muppet character, named Alex, also had an incarcerated parent. Children in the control group watched a general Sesame Street episode on numbers and math, and families were given access to materials after completion of the study. The original study showed promise of these materials, such that the intervention group had more positive visiting in experiences and caregivers also reported positive changes in how they talk to children about the incarceration [39]. Across the waves of data collection, participants also shared information on basic demographics and incarceration-related experiences (such as whether the child witnessed their parents’ crime, arrest, or sentencing).

A targeted sample was enrolled starting with jailed parents who met eligibility criteria. Jailed parents were eligible for participation in the study if they met the following criteria: (1) were at least 18 years old, (2) had a child 3–8 years of age who lived with kin near one of the four the study sites; if parents had more than one child in this age range, one was chosen at random by the study team, (3) retained legal rights to the child, (4) had not committed a crime against the child, (5) cared for the child at least part of the time prior to incarceration (i.e., a resident or engaged parent), (6) did not anticipate being released into the community for at least one week from the date of enrollment, (7) anticipated receiving a visit from the child, and (8) could understand and read English. A total of 284 jailed parents with 3- to 8-year-old children were recruited across four jails in two states, with 86 (30%) child-caregiver dyads participating in the larger study (one focal child per family). Four families were excluded from the present report because the parent had been incarcerated for longer than 180 days, and 6 families were excluded because of missing data in the outcome variables (as the PROCESS macro cannot run using imputed datasets).

Four jail systems from two midwestern states participated in this research; all jails were run by county sheriff’s departments who were in charge of both law enforcement and jails in their counties, and all of the jails had significant racial disparities compared to their county populations. The first jail, from which 21 study participants were enrolled, is located in a large urban community (823-bed capacity, 8000 annual admissions, 788 daily population, 79% men). The second jail, from which 19 study participants were enrolled, is located in an urban community and holds a mix of individuals from urban and rural locations (876-bed capacity, 13,000 annual admissions, 800 daily population, 84% men). The third (*n* = 8) and fourth (*n* = 28) jails are located in suburban regions of a major metropolitan area. The third site is a 200-bed facility for adult men and women that holds pretrial, convicted, and sentenced individuals for up to 365 days or less (average daily population 202, 75% men). The fourth site is a 263-bed facility that detains men only; incarcerated women are transferred to a nearby county.

### 2.3. Measures

Figure 1a shows the approximate timing of the measures administered in the study.

#### 2.3.1. Demographic and Family Characteristics 

Jailed parents completed a Parent Questionnaire inquiring about their demographic characteristics (e.g., age, gender, race, education, marital status, pre-incarceration employment and income), their children (e.g., age and gender of children, exposure to incarceration-related experiences, plans to live with child), parental problems (e.g., drug, alcohol, or mental health problems, treatment for such problems) and the parent’s involvement in the criminal justice system (e.g., arrest and incarceration history, sentence length if known, days in jail for this incarceration).

#### 2.3.2. Witnessing the Parent’s Arrest and Crime 

Children’s incarcerated parents and at-home caregivers were asked questions about the children’s incarceration-related experiences based on Dallaire and Wilson (2010). Children’s incarcerated parents and caregivers were asked whether or not their child witnessed the parent’s arrest and crime and how much distress the child exhibited because of each experience, rated on a scale ranging from 1 (no distress) to 5 (extreme distress). We combined caregivers’ and incarcerated parents’ reports because they may have had access to different information (e.g., not aware of the child’s presence during the crime or arrest). Children were considered as having witnessed the parent’s arrest (or crime) if either the jailed parent or caregiver reported it; for distress ratings, we recorded the higher rating.

To capture both the act of witnessing and the child’s associated distress, we summed two variables: witnessing parental arrest and distress about witnessing parental arrest (*M* = 2.39, *SD* = 2.62). Scores ranged from 0 to 6. Scores of 0 indicated that the child did not witness the parent’s arrest; scores of 1 indicated that the child witnessed the arrest but was not distressed about it. Scores ranging from 2–6 indicated that children witnessed the arrest and showed increasing levels of distress about it. We created a similar variable for witnessing the parent’s crime and distress about it (*M* = 1.67, *SD* = 2.32).

#### 2.3.3. Emotional Reactions to the Parent Leaving for Jail 

Caregivers completed a checklist regarding children’s emotional reactions to separation from the jailed parent used in Poehlmann [53], derived from the work of Hale [62]. The list was presented at the initial data collection and 2 weeks later. The list included 10 common emotions that children exhibit following parental incarceration, including sadness, worry, confusion, anger, fear, depression, embarrassment, guilt, relief, and loneliness [53]. The responses were scored as absent = 0, present = 1, and summed. Guilt and embarrassment were dropped because only two caregivers indicated that children expressed these emotions, likely because of children’s young age. In addition, relief was unrelated to the negative emotions, so it was dropped from the summary score (but still included in the descriptive results). Cronbach’s alpha for the 7 item scale was 0.67 at the initial assessment and 0.68 two weeks later.

#### 2.3.4. Children’s Emotional Symptoms 

Caregiver-report on the Strengths and Difficulties Questionnaire (SDQ) [63,64] was used to capture children’s behaviors that had occurred in the past 6 months. The SDQ is a widely used screening tool appropriate for use with children age 2 to 17 years. It consists of 25 items focusing on behaviors and psychological attributes, some positive and others negative. For the present study we used the SDQ Emotional Symptoms scale, which ranged from 0 to 7 (*M* = 2.53, *SD* = 1.94). Cronbach’s alpha was 0.81.

### 2.4. Child Outcomes

#### 2.4.1. Developmental Milestones 

The PEDS-Developmental Milestones Assessment (PEDS-DM) [65] is a widely used screening measure for children birth to 11 years that assesses children’s developmental milestones. Although items can be completed by parent report or administered directly to children, we administered items directly to children. The PEDS-DM consists of 6–8 items, each tapping into a developmental domain (fine motor, gross motor, expressive language, receptive language, self-help, social-emotional, and for children age 3 and above, early reading and math skills). Not passing an item indicates a missed milestone (i.e., difficulties in that domain), with cutoffs at the 16th percentile and below.

The PEDS-DM has sensitivity and specificity between 70% and 97% across ages and developmental domains. Halle and colleagues [66] found strong correlations between PEDS-DM and other individually-administered standardized test scores. The PEDS-DM has also been used with children of diverse races [67]. Children are considered to have missed a developmental milestone if they do not pass one or more of the items on each scale.

In the present study, first we summed the missed milestones across domains to create a total missed milestones (or total developmental delay) score. Then, to examine developmental domains separately, we created four binary scores that reflected one or more missed milestones in each domain: academic (early literacy, numeracy), social/adaptive (social emotional, adaptive), language (receptive, expressive), and motor (fine, gross). In this sample, 43% of children showed an academic delay, 30% showed a social/adaptive delay, 42% showed a language delay, and 45% showed a motor delay. These rates are similar to those reported for young children with imprisoned mothers (e.g., [53]). 

#### 2.4.2. Child Health

Caregivers reported on children’s overall health using a one-item measure from the National Survey of Children’s Health (NSCH) [68]: “In general, how would you describe this child’s health?” The item was rated on a 1–5 scale, from poor to excellent, with higher numbers indicating better health. Scores ranged from 2–5, with a mean of 4.26 (*SD* = 0.79). Caregivers rated children’s health at the time of the initial assessment and 2 weeks later.

### 2.5. Procedure

Jailed parents and children’s caregivers provided written informed consent for their own and their children’s participation in the research. Research protocols were approved by the Institutional Review Boards at both universities where the research was conducted.

Jailed parents, their children, and their children’s caregivers were enrolled into the larger intervention study from one of four jails [39]. Parents who were in jail reported on their own and children’s background information. Subsequently, data were collected from children and caregivers at the beginning of a visit at the jail. The measures used in this study were administered prior to randomization for the intervention, except for the 2-week follow up measures of child health and children’s emotional reactions to parent’s leaving for jail. The intervention did not have an effect on child health or children’s emotional reactions to the parent leaving for jail, so we included these repeated measures in our analyses.

Trained researchers with experience working with children and families affected by incarceration were responsible for recruitment, enrollment, and assessment of children and caregivers. To accommodate jail operations and policies, slightly different recruitment and enrollment procedures were used across jail sites as described by Poehlmann-Tynan et al., 2021 [39]. Following the consent process, completion of jailed parent questionnaires occurred first in the jail; caregiver questionnaires and child assessments occurred subsequently, during their wait for a visit with the incarcerated parent. Initial data collection with children and caregivers lasted 20–30 min, depending on the wait time for the visit. Caregivers received a phone call 2 weeks later to repeat several measures. Caregivers were paid $50 and children were given stickers and a book following data collection. Jail policies prohibited compensating incarcerated parents for their participation.

### 2.6. Plan of Analysis

Descriptive analyses, Chi-Square tests, paired samples *t*-tests, and McNemar tests were conducted to assess the first three research questions. To address the other research questions, we evaluated a moderated mediation model (Figure 1b) using the PROCESS macro v3.5 [69] executed in SPSS v.26 (IBM Corp: Amork, NY, USA, 2019). Using PROCESS, we examined the indirect path from witnessing the parent’s arrest and associated distress (X) to the outcomes of missed development milestones and caregiver-reported child health (Y), via the mechanism of children’s initial negative emotional reactions to separation from the parent because of parental incarceration (M). We also examined statistical interactions between caregiver-reported child SDQ Emotional Symptoms and witnessing the parent’s arrest (W ∗ X) and SDQ Emotional Symptoms and children’s initial emotional reactions to separation from the parent (W ∗ M).

The PROCESS macro generates 10,000 bootstrapped samples to calculate 95% bias-corrected confidence intervals; interactions that are significant below the *p* < 0.05 level are examined in PROCESS at multiple levels of the moderator and tested for significance. The PROCESS macro also estimates regression coefficients of direct and indirect paths using ordinary least squares regression for continuous outcomes and logistic regression for the binary outcomes, also using 10,000 bootstrapped samples to calculate 95% bias-corrected confidence intervals [69].

The primary set of statistical models included seven analyses in PROCESS, with witnessing parental crime as the key predictor (X): (a) one analysis for total missed developmental milestones as the outcome, (b) one analysis for each of the four PEDS-DM binary scales (academic, language, social/adaptive, and motor) as outcomes, and (c) two analyses for child health (initial assessment and 2 weeks later) as the outcomes. All models controlled for child age, child gender, and parental race (Black = 1, not Black = 0), selected because the variables theoretically related to or were correlated with the outcomes. Additional variables were assessed as covariates but rejected (e.g., incarcerated parent gender; number of prior parental arrests; days parent has been in jail; child’s age at separation from the parent) because they did not relate to the outcomes or were highly correlated with other predictors. Reported effects were characterized as small (*r* = 0.10), moderate (*r* = 0.30), or large (*r* = 0.50) using Cohen’s benchmarks [70].

We ran a second set of PROCESS models adding witnessing parental crime as a covariate. The results of the second set of PROCESS models with witnessing parental crime as a covariate did not substantially differ from the primary models, except for the social/adaptive developmental outcome (as described below). Finally, as a sensitivity analysis, we ran a third set of PROCESS models replacing the witnessing parental arrest variable with the witnessing parental crime variable to ensure that these variables were not interchangeable. For the sensitivity models, the findings were nearly the same as the second set of models with witness crime as a covariate; i.e., there were no statistical interactions between witnessing parental crime and SDQ Emotional Symptoms or children’s emotional reactions to separation from their incarcerated parents. In other words, witnessing parental crime had an effect that was distinct from witnessing parental arrest, and adding witnessing parental crime as a covariate appeared to sufficiently capture this effect. Thus, we report the findings of the primary PROCESS models, with witnessing parental arrest as the key predictor (X), and the second set of PROCESS models, with witnessing parental crime as a covariate.

A power analysis was conducted with G*power 3.1 (Universität Düsseldorf, Germany, https://www.psychologie.hhu.de/fileadmin/redaktion/Fakultaeten/Mathematisch-Naturwissenschaftliche_Fakultaet/Psychologie/AAP/gpower/GPowerManual.pdf, (accessed on 8 April 2021) for the multiple regression and logistic regression analyses used in the PROCESS macro. To detect moderate and large effects in the multiple regression analyses, power was 0.99; to detect small effects, it was 0.77. To detect moderate and large effects in the logistic regression models, power was 0.95 and 0.99, respectively, but only 0.62 to detect small effects.

## 3. Results

### 3.1. Descriptive Research Questions

#### 3.1.1. What Proportion of Children with Jailed Parents Witnessed Their Parent’s Arrest or Crime, and Did This Differ by Parental Race? 

Based on the combination of jailed parent and at-home caregiver reports, 43.4% of the children witnessed their parent’s arrest just prior to the parent’s admission to jail. For the 33 children who witnessed their parent’s arrest, children’s reported distress ranged from 1 to 5, with a mean of 4.23 (*SD* = 1.01), between very and extremely distressed. Three in four young children were reported to be very or extremely distressed when witnessing the parent’s arrest. In addition, 22.4% of the children witnessed their parent’s crime. For the 17 children who witnessed their parent’s crime, children’s reported distress ranged from 0 to 5, with a mean of 3.94 (*SD* = 1.30), between not distressed and extremely distressed. Two in 3 young children were reported to be very or extremely distressed when witnessing the parent’s crime. A paired samples t-test revealed that children were significantly more distressed when witnessing the parent’s arrest compared to witnessing their crime, *t*(75) = 2.075, *p* = 0.041.

A crosstabs analysis indicated that 9 children witnessed both the parent’s arrest and the parent’s crime. Chi-square analysis indicated that children with Black parents were not more likely than other children to witness the parent’s arrest or crime, ***χ***2(1) = 0.002, *p* = 0.96 and ***χ***2(1) = 1.45, *p* = 0.23, respectively. In addition, *t*-tests showed that children’s distress levels regarding witnessing parental arrest or crime were not related to parental race, *t*(74) = −0.66, *p* = 0.51 and *t*(74) = 0.61, *p* = 0.55, respectively.

##### What Emotional Reactions Did Children Exhibit Following the Parent Leaving for Jail, and Did This Change over Time? 

According to their caregivers, children initially exhibited a median of two negative emotional reactions to separation from their parent due to the parent’s incarceration (*M* = 2.22, *SD* = 1.45), with a range from 0 to 6. The most common emotion was sadness, exhibited by 79% of the children; 54% of children exhibited worry, 34% anger, 28% loneliness, and 11% fear. The least common emotion reported was relief (6%).

Two weeks later, children still exhibited a median of two negative emotional reactions to the separation from their parent (*M* = 2.09, *SD* = 1.83), again with a range from 0 to 6. The most common emotion was still sadness, exhibited by 69% of the children; 47% exhibited anger; 45% worry; 24% depression, and 18% fear. In addition, 22% of children expressed relief. Although the total number of children’s emotional reactions did not change over time, paired samples *t*(75) = 1.03, *p* = 0.305, sadness decreased (McNemar test, *p* = 0.013), whereas relief increased (McNemar test, *p* = 0.022). The frequency of other emotions did not significantly change.

### 3.2. Primary PROCESS Models

#### 3.2.1. Predicting Children’s Emotional Reactions to Separation from the Parent Leaving for Jail

##### Initial Emotional Reactions (Time 1)

**Figure 2:**
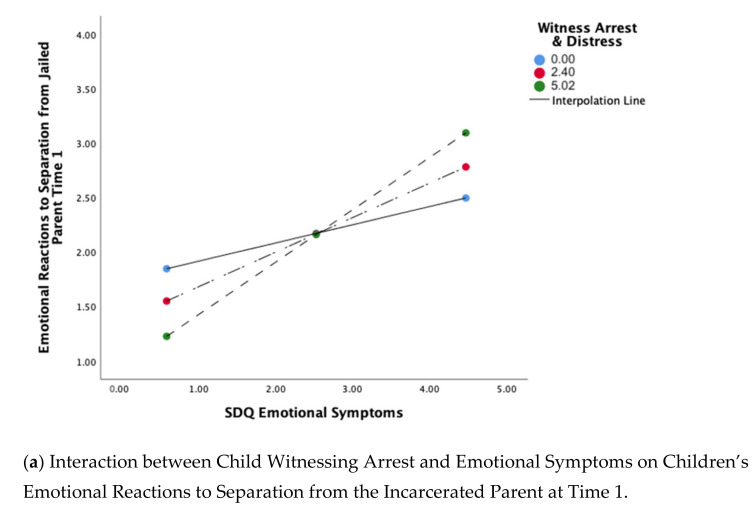

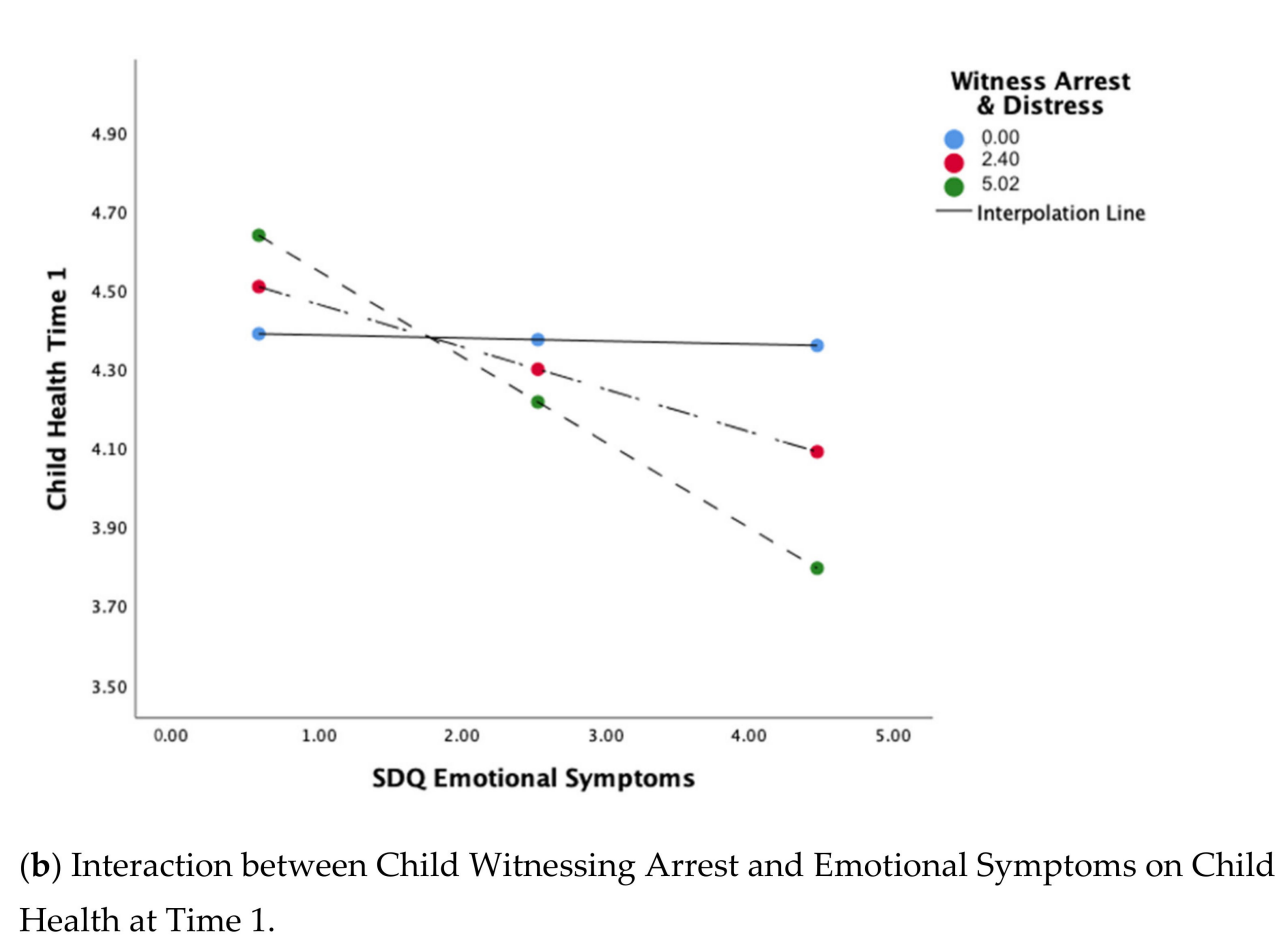
Visual Depiction of Interactions for Children’s Emotional Reactions to Parental Separation and Child Health. *Note. SDQ* = Strengths and Difficulties Questionnaire.

The first analysis in the PROCESS model, with witnessing parental arrest and distress (X) predicting children’s initial emotional reactions to separation from the parent who left for jail (M, the proposed mediator), showed a moderate effect, *R*^2^ = 0.245. The interaction between witnessing the parent’s arrest and SDQ Emotional Symptoms was significant, *p* = 0.040. A test of the interaction indicated that witnessing the parent’s arrest combined with high SDQ Emotional Symptoms predicted more negative emotional reactions to the parent’s departure for jail, *p* = 0.048, but there was no effect of witnessing the arrest at lower levels of SDQ Emotional Symptoms, consistent with our hypothesis (Table 2a; Figure 2a). No other variables were significant predictors.

##### Emotional Reactions at 2 Weeks after the Initial Assessment (Time 2)

We repeated this analysis in PROCESS, with witnessing parental arrest (X) predicting children’s 2-week emotional reactions to the parent leaving for jail. The model showed a small effect, *R*^2^ = 0.151. The SDQ Emotional Symptoms variable was significant, *p* = 0.003, with children high in emotional symptoms also continuing to show more emotional reactions to the separation from their parent because of jail incarceration. No other variables were significant predictors (Table 2b). Two-week emotional reactions were not used further in analyses, as they were deemed similar to the SDQ Emotional Symptoms variable.

#### 3.2.2. Predicting Missed Developmental Milestones as Outcomes

In this section, we present developmental milestone models, first focusing on total delays and then on specific developmental areas.

##### Total Missed Developmental Milestones Model 

The model predicting children’s total missed developmental milestones showed a moderate effect, *R*^2^ = 0.298. There was a significant effect of witnessing the parent’s arrest and associated distress, *p* = 0.018, with higher witness arrest distress scores associated with more missed developmental milestones. There was also a significant interaction between the witness arrest distress variable and SDQ Emotional Symptoms, *p* = 0.043. A test of the interaction indicated that at lower levels of SDQ Emotional Symptoms, witnessing parental arrest and higher distress was associated with more developmental delays, whereas at higher levels of SDQ Emotional Symptoms, witnessing parental arrest and associated distress was not related to total delays (Figure 2b, Table 2c). Child age was significant, *p* < 0.001, with younger children showing more delays than older children. No other variables were significant in this model.

We also examined specific developmental domains in the following analyses:

##### Child Early Academics Models

**Figure 3:**
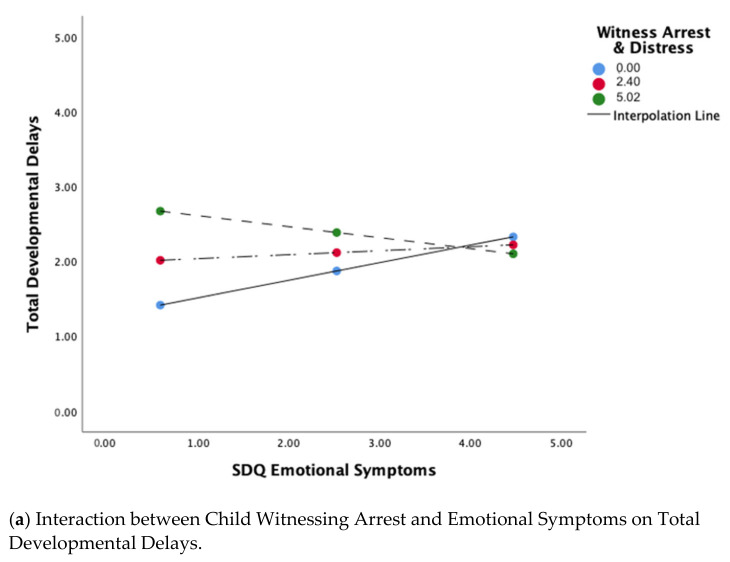

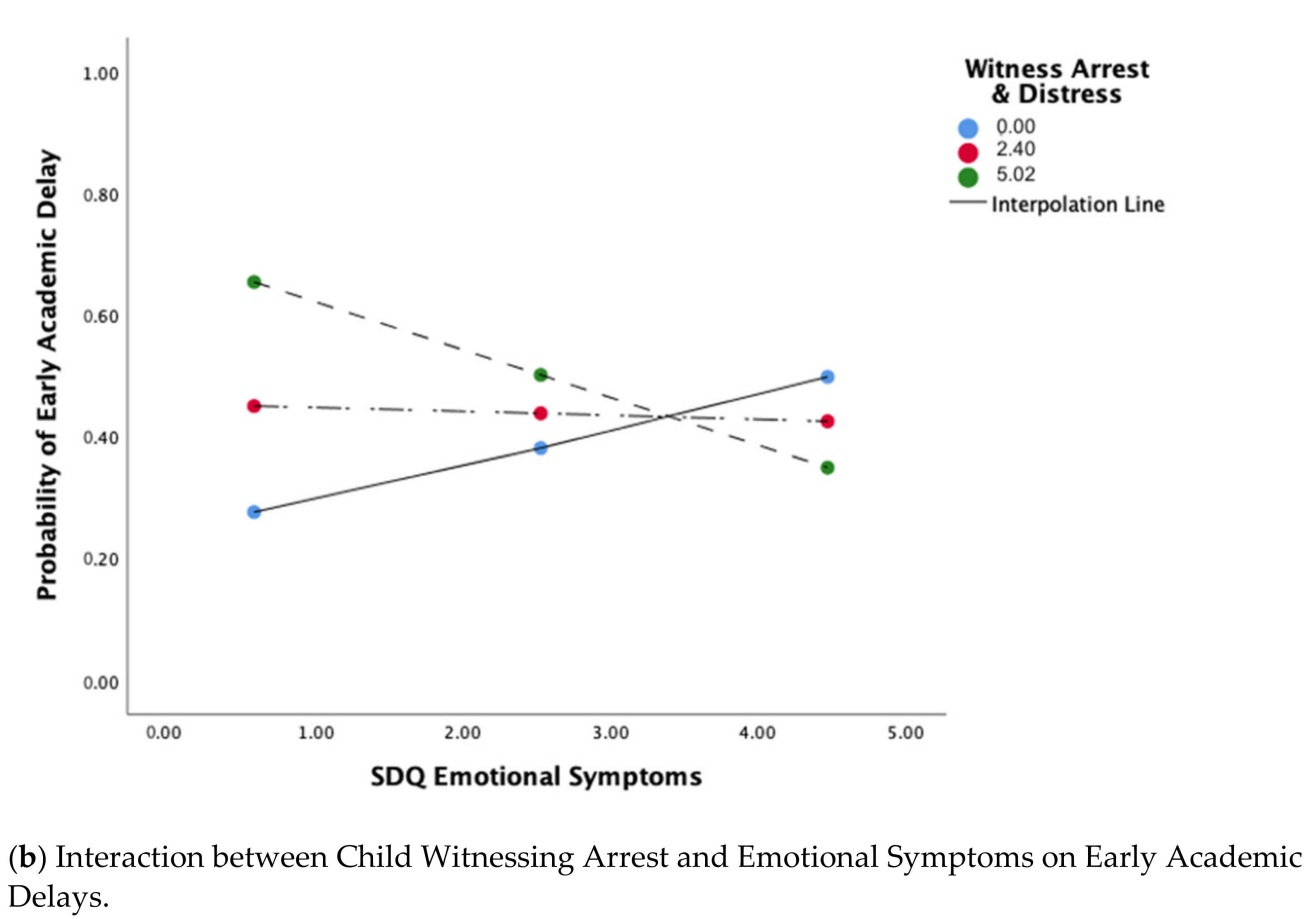
Interaction between Witnessing Parental Arrest and Developmental Outcomes. Note. SDQ = Strengths and Difficulties Questionnaire.

The model predicting children’s early academic skills reflected a small effect overall. However, there was a significant effect of witnessing the parent’s arrest and associated distress, *p* = 0.040, with higher witness arrest distress scores associated with more missed academic developmental milestones. There was also a significant interaction between the witness arrest distress variable and SDQ Emotional Symptoms, *p* = 0.041. A test of the interaction indicated that at lower levels of SDQ Emotional Symptoms, witnessing parental arrest and distress was associated with early academic delays, whereas at higher levels of SDQ Emotional Symptoms, witnessing parental arrest and associated distress was not related to academic delays (Figure 3b, Table 3). Parental race was also significant, *p* = 0.032, with children whose incarcerated parent was Black showing more missed academic milestones than other children. No other variables were significant in this model.

##### Child Language Model

The model predicting children’s language skills reflected a moderate effect, *p* = 0.012, with child age and gender as significant predictors. Younger children and boys were more likely to show developmental delays in their language skills than older children and girls, *p* = 0.009 and *p* = 0.003, respectively. Witnessing parental arrest and other variables were unrelated to child language (Table 3).

##### Child Social/Adaptive Model

The model predicting children’s social/adaptive development also reflected a moderate effect, *p* = 0.024. Child age was a significant predictor of children’s social/adaptive development, *p* = 0.002, with younger children more likely to show missed milestones in the social/adaptive area than older children. No other variables were significant (Table 3).

##### Child Motor Skills Model

The model predicting children’s motor skills reflected a moderate effect, *p* = 0.002. There was a significant effect for SDQ emotional symptoms, *p* = 0.020, with children showing more SDQ emotional symptoms also showing more missed motor milestones. Witnessing the parent’s arrest and associated distress was related to more missed motor milestones at a trend level, *p* = 0.050. There was also a significant effect of child age, *p* < 0.001, with younger children showing more delays than older children (Table 3). No other variables were significant in this model.

#### 3.2.3. Predicting Child Health as Outcome

##### Child Health at Initial Assessment (Time 1) 

The model predicting caregiver-reported child health at time 1 showed a small effect, *R*^2^ = 0.173, although the interaction between witnessing the parent’s arrest and SDQ Emotional Symptoms was statistically significant, *p* = 0.027 (Table 4a). A test of the interaction revealed that at high levels of emotional symptoms, witnessing the parent’s arrest was associated with less optimal child health, *p* = 0.021 (Figure 3), whereas at low emotional symptoms, witnessing the parent’s arrest did not have an effect.

##### Child Health at 2 Weeks after the Initial Assessment (Time 2)

For the child health model at the 2-week follow up, we also included child health at the initial assessment as a predictor. The model predicting caregiver-reported child health at time 2 showed a moderate effect, *R*^2^ = 0.369. Child health at time 1 was a significant predictor, *p* < 0.001, with children who had better health at time 1 also showing better health at time 2 (Table 4b). In addition, the X ∗ M (witnessing arrest ∗ negative emotional reactions to separation) interaction was significant, *p* = 0.017. A test of the interaction indicated that when children showed intense negative emotional reactions to the parent’s initial departure for jail, witnessing the parent’s arrest was associated with less optimal child health at time 2, whereas witnessing the parent’s arrest was unrelated to child health at time 2 when emotional reactions to the parent’s departure were low. Finally, the witnessing parental arrest and distress variable trended toward significance, *p* = 0.075, with children who witnessed parental arrest somewhat more likely to have lower health ratings.

### 3.3. PROCESS Models with Witnessing Parental Crime and Associated Distress as a Covariate

The primary PROCESS models assessed above were re-assessed with the witnessing parental crime and distress variable added as a covariate. Only substantial similarities and differences are reported here, with tables included in the Supplementary Materials upon request from the author.

#### 3.3.1. Emotional Reactions to Separation from the Parent

The results of the analysis predicting children’s initial emotional reactions to separation from the parent (time 1) were virtually identical to the original analysis, *R*^2^ = 0.246. In addition, the analysis of children’s emotional reactions 2 weeks later (time 2) yielded similar findings, although the model was slightly attenuated overall, *R*^2^ = 0.146, with a small effect. The SDQ Emotional Symptoms variable was significant, *p* = 0.006, similar to the primary model.

#### 3.3.2. Missed Developmental Milestones

The model predicting children’s total missed developmental milestones with the witness crime distress variable was nearly identical to the primary analysis, *R*^2^ = 0.311. In addition, the results of the models predicting children’s early academic, language, and motor delays were nearly identical to the original models. However, in the model predicting children’s social/adaptive development, the witnessing parental crime and distress variable was significant, *p* = 0.018, while the effect of child gender was slightly attenuated. When children witnessed their parent’s crime and exhibited distress about it, they were more likely to show missed social/adaptive milestones.

#### 3.3.3. Child Health

For child health at time 1, the model with witness crime as a covariate was similar to the primary model. For child health at time 2, similar results were found as the primary model, *R*^2^ = 0.379, although the witness arrest coefficient was reduced from a trend to non-significance. Child health at time 1 was a significant predictor of child health at time 2, *p* < 0.001, in addition to the interaction between witnessing parental arrest and children’s emotional reactions to separation from their parent (X ∗ M), p = 0.019.

## 4. Discussion

Although witnessing a parent’s arrest may be a relatively common experience for children with incarcerated parents, the experiences of young children who witness parental arrest are largely unexplored. In this multi-method, multi-respondent, within-group short-term longitudinal study, we examined how witnessing parental arrest prior to jail incarceration related to young children’s subsequent health and development, conditional on children’s emotional symptoms. We found that among 3- to 8-year old children with jailed parents, witnessing the parent’s arrest and exhibiting distress about it predicted more missed developmental milestones, especially in early academic skills. In addition, when children had more emotional vulnerabilities, witnessing the parent’s arrest and exhibiting distress about it related to less optimal health at both timepoints, as well as more intense negative emotional reactions to the parent leaving for jail. Children’s reactions to the parent’s departure for jail, conceptualized as a component of their experience of ambiguous loss, functioned as a moderator rather than a mediator of the association between witnessing parental arrest and child outcomes, contrary to our hypothesis. These findings contribute to our understanding of heterogeneity in the experiences of CIP, as well as processes related to child outcomes in the context of parental criminal justice involvement.

### 4.1. Child Overall Health

Child health is understudied in the CIP literature, with only a few published papers contrasting childhood health outcomes for children who have and have not experienced parental incarceration, with few health effects documented [41,42,71,72]. However, health problems related to parental incarceration have been studied more often in adolescents and young adults, with consistent findings (e.g., [45,47]). One explanation for the contrasting findings in early childhood is that studies have not examined within-group variation in health outcomes for young CIP, whereas another explanation has to do with the timing of health assessments relative to when the parent’s incarceration occurred.

In this study, we found conditional associations between witnessing parental arrest and caregiver-reported health in young children with jailed parents following parental jail incarceration. At the initial assessment, witnessing parental arrest and exhibiting more distress about it was associated with less optimal caregiver-reported child health when children’s emotional symptoms such as anxiety and depression were high, but not low. Two weeks later, we found somewhat similar results, with children’s emotional reactions to separation from the jailed parent functioning as a moderator rather than a mediator of the association between witnessing parental arrest and child health. These findings indicate that links between witnessing the parent’s arrest and child health were exacerbated by the young child’s existing emotional concerns and emotional reactions to the jailed parent’s departure, consistent with dual risk [73] or diathesis-stress models (e.g., [74]). The stressors interact, or accumulate, to affect health in children with incarcerated parents.

Recently witnessing a parent’s arrest can be a traumatic experience that has implications for young children’s health, similar to what has been documented in the adult literature regarding the association between adverse childhood experiences and adult health (e.g., [46]). Future population-based longitudinal research should examine how the relation between parental incarceration and children’s health may be conditional on certain risk experiences related to parental criminal justice involvement. Studies should also report how proximal the child’s experiences are relative to the assessment of child health.

### 4.2. Child Social and Emotional Development

When children witnessed their parent’s arrest and showed distress and had high levels of existing emotional symptoms—such as anxiety, withdrawal, and depression—they not only exhibited less optimal health but also more initial negative emotional reactions to their parent’s departure for jail, which was conceptualized as a reflection of feelings related to ambiguous loss. In turn, children’s initial emotional reactions to their parent’s departure, combined with witnessing the parent’s arrest, predicted child health at time 2, over and above the effects of child health at time 1.

For young children, the distress-inducing and potentially traumatic experience of witnessing the parent’s arrest appeared to exacerbate children’s pre-existing tendencies toward anxiety, withdrawal and depression, contributing to intensified experiences of loss following parental incarceration. At the 2-week follow up, the only significant predictor of children’s time 2 emotional reactions to separation from their parent was their emotional symptoms, as measured by the Strengths and Difficulties Scale screener. As time goes by and the parent-child separation continues, it is possible that young children’s initial reactions to separation from the parent and reactions to witnessing parental arrest may become indistinguishable from their general anxiety, worry or depression or incorporated into how their caregivers perceive their overall health. Such processes may also occur later in development. For example, Turney [33] found that during adolescence, personal contact with police as well as vicarious contact with police was related to more depressive symptoms, especially when police contact was intrusive. Such findings have implications for the health and mental health of CIP and interventions, as discussed below.

Children’s common initial emotional reactions following separation from their resident or engaged parent, as reported by children’s at-home caregivers, included sadness, worry, anger, and loneliness. These reactions are similar to previous studies of young children with incarcerated parents (e.g., [53]). Two weeks later, however, children in the present study were reported by caregivers to exhibit less sadness but more relief as a result of their parent’s departure for jail. We do not have the data to determine why some children felt relief—perhaps the situation had stabilized, or children knew their parent was safe because they had visited with their incarcerated parent by that time, or the parent had engaged in behavior at home that was negative and it had stopped with their departure for jail. Future studies should explore these possibilities, as potentially positive effects of parental incarceration are rarely reported in the literature, other than research examining child well-being when incarcerated fathers had severe substance abuse [75].

As far as children’s achievement of social and adaptive developmental milestones was concerned, children’s age was the strongest predictor, with younger children showing more social and adaptive developmental delays. In addition, children were more likely to exhibit missed social and adaptive milestones when they had witnessed their parent’s crime and expressed distress about it, but not their parent’s arrest. Social learning theories suggest that parental modeling can affect children’s social development (e.g., [76])—perhaps in young children whose parents modeled criminal behavior in front of them, children lagged behind in certain social milestones. It should also be noted that there may be different effects of witnessing a parent’s crime when it does not lead to arrest and incarceration, and all of the children in this study experienced parental incarceration.

Children’s social emotional development and adaptive skills are important foundations for resilience. Social skills are being taught in classrooms to promote resilience (for a review, see [77]) and this should be examined with more care in future research with CIP. In the present study, it appeared that social and adaptive development were relative strengths for young children with jailed parents, as children showed fewer missed milestones in these areas than in other developmental areas. Social and adaptive development may be good candidates in future research examining protective or promotive factors in children with incarcerated parents.

### 4.3. Early Childhood Cognitive Development in the Context of Parental Incarceration

Population-based studies of CIP have found that parental incarceration is associated with less optimal child, adolescent, and young adult cognitive and educational outcomes, including developmental delays, learning disabilities, special education placement, grade retention, suspension, and expulsion [5,37,38,42,78,79]. For instance, parental incarceration has been found to significantly reduce school readiness in preschoolers [80] and that teen’s vicarious contact with police negatively influences academic achievement [31]. However, only a few studies have explored mediators or moderators of the relation between paternal incarceration and child educational outcomes (e.g, [81]. Although toxic stress has not yet been measured in CIP, Haskins [38] has suggested that direct and indirect exposure to trauma, such as witnessing parental arrest or residing in a violent neighborhood, could lead to less optimal cognitive development in CIP via the mechanism of toxic stress.

Even though we did not directly measure toxic stress in this study as has previously been done [20], we examined how a stressful experience--witnessing the parent’s arrest--related to missed developmental milestones in early childhood, including academic delays, following a parent leaving for jail. We found that witnessing the parent’s arrest and exhibiting distress about it predicted more missed developmental milestones, especially in early academic skills. In addition, there was an interaction between witnessing the arrest and children’s total developmental delays and academics. Specifically, when their general emotional symptoms were low, children who witnessed their parent’s arrest were more likely to miss early developmental milestones, especially in the areas of early literacy and numeracy skills. Thus, unlike our health and emotional development findings, a dual risk or diathesis stress effect was not present for overall developmental delays and academic milestones.

The effects of witnessing a parent’s arrest may be particularly obvious for early childhood developmental and academic skills when children show low emotional symptoms overall. Although it may seem counterintuitive, such heterogeneity has also been documented in children with incarcerated fathers [72] and mothers [82]. Indeed, children who are least likely to experience parental incarceration (i.e., the least likely to experience the types of risks that “select” one into the parental incarceration group) are the ones who appear most affected by it. The findings emphasize how the magnitude of effect may be greater for those without as many other vulnerabilities and thus more likely to be detected (or attributed to the parent’s incarceration). Children may still be highly distressed by their experiences related to parental incarceration or witnessing the parent’s arrest, even though they have not experienced many other risk factors or emotional vulnerabilities.

Stress associated with witnessing a parent’s arrest may disrupt cognition and problem solving in otherwise emotionally adapted young children (Shonkoff et al. 2012), thus affecting early learning and skills associated with school readiness. Children who were already struggling with elevated emotional symptoms, such as anxiety, withdrawal or depression, were less impacted by witnessing the arrest or crime with respect to their early developmental milestones, especially early literacy and numeracy, unlike their health outcomes, which were impacted more by witnessing parental arrest in the context of emotional vulnerabilities. In future research, mechanisms related to toxic stress should be examined in the context of CIP witnessing their parent’s arrest and other trauma, especially in relation to their academic and health outcomes to determine specific pathways of effects. For example, it is possible that certain executive functions may be disrupted by specific traumatic events, such as witnessing or experiencing a parent’s arrest, whereas other executive skills may not be affected (e.g., [83]). In addition to examining academic outcomes in children and adolescents with incarcerated parents, more attention should be paid to underlying cognitive and attentional mechanisms that are particularly sensitive to the effects of acute and chronic stress in early development (e.g., [21]).

We included parental race in our models because Black parents are overrepresented in the criminal justice system as a result of systemic racism, and also because of the possibility that children may have witnessed violence toward their Black parents during arrest [61]. Although children with incarcerated Black parents were not more likely to witness the parent’s crime or arrest or exhibit different levels of distress then other children in this study, parental race significantly predicted children’s early academic skills. Children of Black jailed parents showed more academic delays than other children, possibly because of stress, unequal treatment in school, or challenges with material resources at home or in their neighborhoods [84]. Stress could also have resulted from early experiences of racism or to witnessing more violent parental arrests. Our finding is consistent with previous research in the CIP literature. For example, using individual-effects models to estimate population level effects, Haskins [38] suggests that paternal incarceration explains 2–15% of the Black–White school achievement gap by middle school. Similarly, Wakefield and Wildeman [6] estimate that racial disparities in parental incarceration are responsible for increases in Black–White inequality in the U.S. ranging from 5–10%. Their statistical overrepresentation in this study, as in the general population of children with incarcerated parents (e.g., [5,85]), suggests that Black children may be more likely than other children to be exposed to incarceration-related events at the population level, such as witnessing parental arrest, contributing to stress. Although young children were equally exposed to witnessing the parental arrest and crime and were equally likely to show distress about it, our data unfortunately were not able to determine if Black children were exposed to more police violence during the parent’s arrest. This should be examined in future studies focusing on children witnessing their parent’s arrest or arrests that occur in the home or neighborhood.

### 4.4. Child Language and Motor Skills

For children’s early language and motor skills, there was a large effect of child age, with younger children being more likely to miss milestones than older children. Younger CIP may be more vulnerable to experiencing adversity than older children [55], which may affect their stress levels and development. It is also possible that as children grow older and attend school regularly, they may “catch up” in some of their skills. For motor skills, higher emotional symptoms were also related to more missed motor milestones, suggesting overlap in children’s developmental domains.

In analyses of data from the Fragile Families and Child Wellbeing (FFCW) study, findings about children’s language development have been mixed. Children who experienced paternal incarceration scored lower on one-word receptive vocabulary tests compared to their peers who never experienced paternal incarceration, whereas those who experienced maternal incarceration did not score lower than peers without incarcerated parents [37,82]. In addition, Dallaire and Wilson’s [19] study with children aged 7–17 found that witnessing incarceration-related events (i.e., a parent’s arrest and crime) was associated with less optimal outcomes for CIP, including their one-word receptive vocabulary. We did not find an effect of witnessing the parent’s arrest or crime on children’s language milestones, although the children in our study were younger than in the Dallaire and Wilson [19] study. Given these different results, the pathways between parental incarceration and child language development should be examined further in CIP at different ages.

### 4.5. Limitations

The present study has numerous limitations that should be considered when interpreting the findings. First, the study used a targeted sample of young children with co-resident or engaged parents who were in jails in areas without child-sensitive arrest protocols. Thus, the results are not population-based; the findings are not generalizable to the entire population of young children with jailed parents, to children with imprisoned parents, to younger or older CIP, to children who had no connection with their incarcerated parents, and to families who live in areas where there are child-sensitive arrest protocols in place. Second, parental arrest does not directly lead to incarceration in every case [86], and this study does not represent families in which a child witnessed the parent’s arrest but the parent was not subsequently incarcerated. Similarly, while study exclusion criteria prevented crimes that parents were arrested for from being against the child or the family, limited context around the crime and subsequent arrest were garnered. Future work in this area should explore the nuances behind this more in order to understand how degree of offense (i.e., violent vs. non-violent) may play into these associations. Third, we collected new data instead of using an existing longitudinal data set with a nationally representative or population-based sample because existing studies have not measured constructs that we thought were important to study in young children with parents involved in the criminal justice system (e.g., [87]), thus leading to a small sample size. Because of the small sample size, we had power to detect moderate and large effects only; because some of the effects appeared to be small, future studies should ensure that samples are large enough to detect small effects. Fourth, although having access to triadic data is a unique aspect of this study, many of the families had complex structures. Some families had multiple adults involved in children’s care and we did not interview more than one at-home caregiver. Similarly, another weakness of this study is that most of the measures relied on caregiver and parent report, though our use of direct assessment of children’s development is a strength. Indeed, because incarcerated parents may be under significant stress and structural constraints, meanwhile also facing stigma that may prevent them from disclosing every challenge that their families face, it may be important to use alternative sources of information to corroborate results. Next, we were unable to control for pre-incarceration development and learning in children, other than asking caregivers to rate children’s emotional symptoms during the past 6 months. Additionally, it may be important for future work to include a more specific measure of the timing between witnessing arrest and the outcomes of interest so as to better isolate the role of arrest in influencing children’s adjustments. Finally, we did not directly measure children’s perceived or physiological stress as a function of whether or not they witnessed the parent’s arrest or crime. Future work should include physiological measures that directly capture young children’s stress responses to parental incarceration-related events or involvement in the criminal justice system.

### 4.6. Implications for Criminal Justice System Policy and Practice

Our findings have implications for law enforcement, corrections, and criminal justice system policy and practice. The study provides evidence of adverse health and emotion outcomes for children when they witness their parents’ arrest in the context of emotional vulnerability, calling for immediate action on the part of law enforcement to increase training on how to prevent trauma in children during parental arrest. We recommend that agencies that have yet to implement safeguarding strategies for parental arrests do so immediately, particularly with regard to implementing prearrest planning to determine if children are present, removing children from sight, refraining from using force, and allowing for the parent to speak with the child prior to detainment to mitigate risk of ambiguous loss and additional trauma. Use of such techniques to protect children could also lead to less violence during arrests in general, and with Black parents in particular. For example, in Minneapolis Minnesota, a police officer shot and killed Philando Castile while he was sitting in the driver’s seat of his car, after being pulled over for a suspected traffic violation. Mr. Castile’s stepdaughter was sitting in the back seat of the car and witnessed the entire event. Had officers used child-sensitive protocols, a rapid escalation of events and such extreme use of force may have been less likely.

Prevention and intervention scientists, as well as attorneys, have similarly urged law enforcement to adopt child-sensitive frameworks such as *First, Do No Harm* [25] and *REACT* [88]. These frameworks emphasize the importance of modifying arrest procedures when children are present, adapting protocols to account for the care of children after adult arrests, and collaborating with professionals for follow-up services to prevent subsequent negative outcomes for children. The extent to which these policies can be widely adopted, implemented, and monitored is likely associated with reducing risk and trauma exposure that has effects on children, parents, and families.

Following a parent’s arrest and detainment, jails have the potential to support children and families [4]. Jail programming that supports parent-child communication and child-friendly contact can be implemented to buffer the impact of parental incarceration and witnessing parental arrest on child development and help decrease feelings of loss. Corrections policies should also tailor programs and services to the needs of incarcerated parents and their children. Pointedly, children remain at the forefront of the Urban Institute’s toolkit [89], designed for developing corrections and community services that strengthen parent-child bonds, promote positive interactions with children, and give parents more decision-making power. Programs for mothers [90] and fathers [91] that are usually administered in prison can be adapted to jails, including components focusing on how to hold developmentally appropriate conversations with children about the parent’s arrest and incarceration, along with reentry strategies that reduce the risk of parental re-arrest. Additionally, more recent work has been done to develop alternatives to incarceration for justice-involved parents (e.g., [92]). This work suggests that there may be safeguarded benefits of offering parents convicted of certain offenses (as of now this work is largely concentrated among those charged with low-level, non-violent offenses) community-based programming in lieu of jail or prison time. These efforts may indirectly improve child outcomes by mitigating certain risks of re-incarceration or elements of household dysfunction (i.e., in the case of drug treatment programs or mental health services) as well as directly by increasing the amount of exposure that a child may have with their parent.

Outside of law enforcement and corrections, the findings have implications for professionals interacting within children’s ecological systems when parents are involved in the criminal justice system. Professionals from pediatricians to childcare providers and teachers to child welfare workers should be aware that many young children with incarcerated parents show delayed developmental milestones and are likely to have experienced trauma. This is particularly important for social workers who make placement decisions for children immediately following their parent’s arrest. Adults should talk with children in developmentally appropriate language, and answer children’s questions simply and truthfully, and parents—even those who are being detained—should be included in decision-making processes. In recognition of the detrimental health consequences of witnessing traumatic events, especially in the context of children’s emotional vulnerability, referrals to health care professionals and mental health providers who are trained in childhood trauma should include information about whether the child was present for the parent’s arrest or crime so they can help children cope with feelings of loss and stress and prevent both short- and long-term emotional and health consequences. While connecting a child to community services can be protective for children, referrals to early childhood education services can also be helpful, especially noting the high rates of young children with incarcerated parents who are lagging on their achievement of developmental milestones. Given this observation, we also recommend that health care providers screen CIP for developmental delays. Linking children to services in their communities or schools is an approach that can help, not only for health and social emotional well-being, but also to support children’s learning outcomes, cognitive development, and problem solving in the future. Indeed, this study has implications for programs that facilitate the development of preacademic skills and early childhood social development (e.g., Head Start or prekindergarten; after school or summer programs). Moreover, referring criminal justice involved families with young children to multigenerational interventions, such as the 2-generation Triple P program, or other programs that also include grandparents, may also be helpful for children’s development. Family home visiting programs may play a significant role in this, as well. For instance, home visiting programs have been found to alleviate harm and improve well-being and learning outcomes, meanwhile bolstering resilience [93,94].

A substantial body of evidence indicates that toxic stress functions as a causal link between early childhood adversity—including trauma and violence exposure—and compromised child development (for a review, see [21]). This work has only recently been applied to children who witness their parents’ arrest [20]. Future work focusing on children with incarcerated parents should assess children’s physiological stress in addition to their behavioral and emotional stress reactions following witnessing a parent’s arrest or crime, or other violence exposures, to further test this mechanism. Toxic stress, when experienced in early childhood, can have lifelong effects on brain development, emotions, social skills, and learning [21]. However, there is heterogeneity in the effects of toxic stress on child development and neural plasticity, and children’s cognitive development and executive functions, when preserved--especially in the context of stable and supporting caregiving--may become protective factors for their development as they move into middle childhood and adolescence. For children with parents in the criminal justice system, with its overrepresentation of people of color because of long-term systemic racism, protective factors related to extended family, community, and church may be particularly important for their development [95]. Such protective factors should be explored in future research, especially longitudinal studies with population-based samples.

## 5. Conclusions

In sum, many young children with jailed parents have witnessed their parent’s arrest, which is often a distressing, even traumatic experience for young children in the absence of child-sensitive arrest protocols. When children are emotionally vulnerable, witnessing the parent’s arrest is related to less optimal child health and more intense negative emotional reactions to separation from an engaged or co-resident parent who goes to jail. Even when children do not exhibit such emotional vulnerabilities, witnessing a parent’s arrest with accompanying high levels of distress relates to children’s missed developmental milestones, especially in the area of early academic learning. These findings are particularly concerning given the high rates of arresting and jailing people in the United States, most of whom are likely to be parents. Interventions designed to take a social justice approach to reducing system-induced risk and supporting children’s strengths and family resilience processes are important across all phases of a parent’s criminal justice involvement, but particularly at the time of parental arrest.

## Figures and Tables

**Table 1:** Jailed Parent Characteristics (N = 76).

Variable		*n*%/Mean (SD)	Range
Race and Ethnicity			
	White	30 (39%)	
	Black/African American	28 (38%)	
	Native American	4 (5%)	
	Latinx	10 (13%)	
	Multiple races/ethnicities	4 (5%)	
Education			
	Some high school or less	16 (21%)	
	High school graduate or equivalency	36 (47%)	
	Partial college or specialized training	22 (29%)	
	College graduate or higher	2 (3%)	
Marital Status			
	Separated or divorced Never married, not partnered Married or partnered	25 (33%) 45 (59%) 6 (8%)	
Employed in the month before arrest		37 (49%)	
Previous incarceration		74 (97%)	
Drug or alcohol treatment		60 (79%)	
Mental health treatment		30 (39%)	
Plan to live with child upon release		64 (84%)	
Age (in years)		32.6 (8.5)	18–53
Monthly income (in dollars)		876.6 (1373.4)	0–8000
Total prior arrests		13.6 (16.6)	0–143
Time served (in days)		46.28 (41.39)	1–170

**Table 2:** Multiple Regression Results from PROCESS Models: Emotional Reactions to Separation and Total developmental Delays.

**(a) Predictors of Young Children’s Emotional Reactions to Parents Leaving for Jail, Time 1 (N = 76).**						
**Predictor**	***β***	***SE***	***t***	**95% CI**		***p***
				**LL**	**UL**	
Constant	1.853	0.614	3.017	0.628	3.078	0.004
Witness Arrest (X)	−0.160	0.103	−1.545	−0.366	0.047	0.127
SDQ Emotional Symptoms (W)	0.167	0.105	1.594	−0.042	0.376	0.115
X ∗ W	0.062	0.030	2.092	0.003	0.122	0.040
Child Gender	−0.005	0.087	−0.059	−0.180	0.169	0.953
Child Age	0.258	0.307	0.841	−0.354	0.870	0.403
Jailed Parent Race	−0.471	0.310	−1.519	−1.089	0.148	0.133
Model Summary *R*^2^ = 0.245, *F*(6,69) = 3.737, *p* = 0.003						
**(b) Predictors of Young Children’s Emotional Reactions to Parents Leaving for Jail, Time 2 (N = 76).**						
**Predictor**	***β***	***SE***	***t***	**95% CI**		***p***
				**LL**	**UL**	
Constant	0.053	0.812	1.296	−0.568	2.674	0.199
Witness Arrest (X)	0.137	0.517	0.607	−0.343	0.294	0.202
SDQ Emotional Symptoms (W)	0.397	0.138	2.865	0.120	0.673	0.006
X ∗ W	−0.023	0.039	−0.586	−0.102	0.056	0.560
Child Gender	0.046	0.406	0.114	−0.764	0.857	0.909
Child Age	0.005	0.116	0.045	−0.226	0.236	0.965
Jailed Parent Race	0.075	0.410	0.182	−0.743	0.892	0.856
Model Summary *R*^2^ = 0.143, *F*(6,69) = 3.074, *p* = 0.089						
**(c) Predictors of Young Children’s Total Developmental Delays (N = 76).**						
**Predictor**	***β***	***SE***	***t***	**95% CI**		***p***
				**LL**	**UL**	
Constant	3.979	0.800	4.974	2.382	5.575	0.000
Witness Arrest (X)	0.294	0.122	2.417	0.051	0.537	0.018
Emotional Reactions to Parents Leaving (M)	−0.020	0.214	−0.094	−0.447	0.407	0.925
SDQ Emotional Symptoms (W)	0.217	0.180	1.201	−0.143	0.576	0.234
X ∗ W	−0.076	0.037	−2.067	−0.149	−0.003	0.043
M ∗ W	0.008	0.067	0.121	−0.125	0.141	0.904
Child Gender	−0.589	0.356	−1.655	−1.299	0.121	0.103
Child Age	−0.455	0.101	−4.525	−0.656	−0.255	0.000
Jailed Parent Race	0.299	0.363	0.822	−0.427	1.024	0.414
Model Summary *R*^2^ = 0.298, *F*(8,67) = 3.549, *p* = 0.002						

Note. CI = Confidence Interval; LL = Lower Level; UL = Upper Level; SDQ = Strengths and Difficulties Questionnaire.

**Table 3:** Logistic Regression Results from PROCESS Models: Specific Developmental Delays.

	**Child Academic Delay**						**Child Language Delay**					
**Variables**	***B***	***S.E.***	**z**	**95% CI**		***p***	***B***	***S.E.***	**z**	**95% CI**		***p***
				**LL**	**UL**					**LL**	**UL**	
Constant	−0.486	1.168	−0.416	−2.774	1.803	0.678	2.226	1.289	1.726	−0.301	4.753	0.084
Witness Arrest (X)	0.385	0.188	2.047	0.016	0.753	0.041	0.174	0.182	0.958	−0.182	0.531	0.338
Emotional Reactions to Parent Leaving (M)	0.113	0.330	0.342	−0.534	0.759	0.733	0.277	0.334	0.830	−0.377	0.931	0.407
SDQ Emotional Symptoms (W)	0.152	0.270	0.562	−0.378	0.681	0.574	−0.058	0.290	−0.199	−0.625	0.510	0.843
X ∗ W	−0.114	0.055	−2.050	−0.222	−0.005	0.040	−0.086	0.057	−1.505	−0.198	0.026	0.132
X ∗ M	0.042	0.099	0.429	−0.151	0.236	0.668	0.029	0.109	0.266	−0.185	0.243	0.791
Child Gender	−0.117	0.509	−0.230	−1.115	0.881	0.818	−1.738	0.587	−2.961	−2.888	−0.587	0.003
Child Age	−0.236	0.148	−1.593	−0.526	0.054	0.111	−0.448	0.172	−2.601	−0.786	−0.110	0.009
Jailed Parent Race	1.138	0.532	2.138	0.095	2.181	0.032	0.423	0.556	0.760	−0.668	1.513	0.447
	Model Summary *χ*2(8) = 11.694, *p* = 0.165 *R*^2^_McFadden_ = 0.112, *R*^2^_Cox-Snell_ = 0.143, *R*^2^_Nagelkirk_ = 0.191						Model Summary *χ*2(8) = 19.609, *p* = 0.012 *R*^2^_McFadden_ = 0.190, *R*^2^_Cox-Snell_ = 0.227, *R*^2^_Nagelkirk_ = 0.306					
**Child Social Adaptive Delay**							**Child Motor Delay**					
**Variables**	***B***	***S.E.***	**z**	**95% CI**		***p***	***B***	***S.E.***	**z**	**95% CI**		***p***
				**LL**	**UL**					**LL**	**UL**	
Constant	2.995	1.378	2.173	0.293	5.696	0.030	1.779	1.275	1.395	−0.721	4.279	0.163
Witness Arrest (X)	0.173	0.197	0.882	−0.212	0.559	0.378	0.394	0.201	1.961	0.050	0.000	0.788
Emotional Reactions to Parent Leaving (M)	−0.334	0.390	−0.855	−1.099	0.431	0.392	0.046	0.375	0.124	−0.688	0.780	0.902
SDQ Emotional Symptoms (W)	−0.003	0.322	−0.008	−0.633	0.628	0.993	0.755	0.324	2.329	0.120	1.39	0.020
X ∗ W	0.008	0.062	0.125	−0.114	0.130	0.900	−0.088	0.059	−1.490	−0.203	0.028	0.136
X ∗ M	−0.016	0.134	−0.121	−0.279	0.247	0.904	−0.098	0.113	−0.865	−0.319	0.124	0.387
Child Gender	−0.878	0.604	−1.454	−2.061	0.306	0.146	−0.015	0.561	−0.027	−1.115	1.085	0.978
Child Age	−0.590	0.191	−3.090	−0.964	−0.216	0.002	−0.696	0.190	−3.662	−1.068	−0.323	0.000
Jailed Parent Race	−0.150	0.577	−0.260	−1.280	0.980	0.795	0.067	0.567	0.118	−1.044	1.177	0.906
	Model Summary *χ*2(8) = 17.662, *p* = 0.024 *R*^2^_McFadden_ = 0.189, *R*^2^_Cox-Snell_ = 0.207, *R*^2^_Nagelkirk_ = 0.293						Model Summary *χ*2(8) = 25.054, *p* = 0.002 *R*^2^_McFadden_ = 0.240, *R*^2^_Cox-Snell_ = 0.281, *R*^2^_Nagelkirk_ = 0.376					

Note. CI = Confidence Interval; LL = Lower Level; UL = Upper Level; SDQ = Strengths and Difficulties Questionnaire.

**Table 4:** Multiple Regression Results from PROCESS Models: Child Health.

**(a) Predictors of Young Children’s Health at Time 1 (N = 76).**						
**Predictor**	***β***	***SE***	***t***	**95% CI**		***p***
				**LL**	**UL**	
Constant	4.466	0.400	11.161	3.668	5.265	0.000
Witness Arrest (X)	0.074	0.061	1.210	−0.048	0.195	0.231
SDQ Emotional Symptoms (W)	−0.014	0.090	−0.157	−0.194	0.166	0.876
X ∗ W	−0.042	0.018	−2.263	−0.078	−0.005	0.027
M ∗ M	0.003	0.033	0.087	−0.064	0.069	0.931
Child Gender	−0.010	0.178	−0.058	−0.366	0.345	0.954
Child Age	−0.069	0.050	−1.366	−0.169	0.032	0.176
Jailed Parent Race	0.121	0.182	0.664	−0.242	0.484	0.509
Model Summary *R*^2^ = 0.173, *F*(8,67) = 1.758, *p* = 0.101						
**(b) Predictors of Young Children’s Health at Time 2 (N = 76).**						
**Predictor**	***β***	***SE***	***t***	**95% CI**		***p***
				**LL**	**UL**	
Constant	2.418	0.639	3.785	1.142	3.694	0.001
Witness Arrest (X)	−0.105	0.058	−1.806	−0.221	0.011	0.075
Emotional Reactions to Parents Leaving (M)	0.003	0.102	0.029	−0.201	0.207	0.977
SDQ Emotional Symptoms (W)	−0.053	0.085	−0.627	−0.223	0.117	0.533
X ∗ W	0.030	0.018	1.639	−0.006	0.065	0.106
X ∗ M	−0.022	0.031	−0.702	−0.085	0.041	0.485
Child Gender	0.014	0.168	0.085	−0.321	0.350	0.933
Child Age	−0.068	0.048	−1.414	−0.164	0.028	0.162
Jailed Parent Race	0.117	0.184	0.636	−0.251	0.486	0.527
Child Health at Time 1	0.557	0.115	4.831	0.327	0.787	0.001
X ∗ M *F*(1,65) = 6.009, *p* = 0.017						
Model Summary *R2* = 0.369, *F*(9,66) = 4.295, *p < 0*.001						

Note. CI = Confidence Interval; LL = Lower Level; UL = Upper Level; SDQ = Strengths and Difficulties Questionnaire.

## Data Availability

The de-identified data set is available from the corresponding author upon request.

## Supplementary Materials

The following are available online at https://www.mdpi.com/article/10.3390/ijerph18094512/s1.
