# Supplementary material for: The Health and Development of Young Children Who Witnessed Their Parent’s Arrest Prior to Parental Jail Incarceration
Source: Int J Environ Res Public Health. 2021 Apr 23;18(9):4512. doi: 10.3390/ijerph18094512 (PMC8123032; doi:10.3390/ijerph18094512)
Supplement: Supplementary file 1 [file ijerph-18-04512-s001.zip › ijerph-1166610-SI.pdf]

Supplementary Table 1 (Compare to Table 2). *Multiple Regression Results from PROCESS Models: Emotional Reactions to Separation and Total Developmental Delays.*

a. *Predictors of Young Children's Emotional Reactions to Parents Leaving for Jail, Time 1* (*N*=76).

Predictor	***β***	*SE*	*t*	95% CI LL	95% CI UL	*p*
Constant	1.855	0.612	3.029	0.633	3.077	0.003
Distress (X)	-0.204	0.126	-1.628	-0.455	0.046	0.108
SDQ Emotional Symptoms (W)	0.167	0.105	1.598	-0.042	0.376	0.115
X * W	0.076	0.036	2.110	0.004	0.148	0.039
Child Gender	0.261	0.307	0.849	-0.352	0.873	0.399
Child Age	-0.004	0.088	-0.044	-0.178	0.171	0.965
Jailed Parent Race	-0.457	0.310	-1.476	-1.075	0.161	0.145
Model Summary $R^2 = 0.245$, $F(6,69) = 3.742$, $p = 0.003$						

b. *Predictors of Young Children's Emotional Reactions to Parents Leaving for Jail, Time 2 (N=76)*

Predictor	***β***	*SE*	*t*	95% CI LL	95% CI UL	*p*
Constant	1.080	0.811	1.333	-0.537	2.698	0.187
Distress (X)	0.066	0.166	0.399	-0.266	0.398	0.691
SDQ Emotional Symptoms (W)	0.391	0.139	2.822	0.115	0.668	0.006
X * W	-0.024	0.048	-0.504	-0.119	0.071	0.616
Child Gender	0.049	0.406	0.120	-0.762	0.860	0.905
Child Age	0.006	0.116	0.050	-0.225	0.237	0.960
Jailed Parent Race	0.070	0.410	0.172	-0.747	0.888	0.864
Model Summary $R^2 =0.143$, $F(6,69) = 1.905$, $p = 0.092$						

c. *Predictors of Young Children's Total Developmental Delays* (*N*=76).

Predictor	***β***	*SE*	*t*	95% CI LL	95% CI UL	*p*
Constant	3.993	0.799	4.995	2.397	5.588	0.000
Distress (X)	0.356	0.148	2.401	0.060	0.652	0.019
Emotional Reactions to Parents Leaving (M)	-0.014	0.214	-0.065	-0.442	0.414	0.949
SDQ Emotional Symptoms (W)	0.217	0.180	1.200	-0.144	0.577	0.234
X * W	-0.090	0.045	-2.026	-0.179	-0.001	0.047
M * W	0.006	0.067	0.091	-0.127	0.139	0.928
Child Gender	-0.589	0.356	-1.654	-1.300	0.122	0.103
Child Age	-0.457	0.101	-4.540	-0.659	-0.256	0.000
Jailed Parent Race	0.276	0.363	0.761	-0.449	1.001	0.450
Model Summary $R^2 = 0.297$, $F(8,67) = 3.535$, $p = 0.002$						

Note. *CI* = Confidence Interval; *LL* = Lower Level; *UL* = Upper Level; *SDQ* = Strengths and Difficulties Questionnaire

Supplementary Table 2 (Compare to Table 3). *Logistic Regression Results from PROCESS Models: Specific Developmental Delays.*

Child Academic Delay

Variables	*B*	*S.E.*	z	95% CI LL	95% CI UL	*p*
Constant	-0.388	1.152	-0.336	-2.646	1.871	0.737
Distress (X)	0.414	0.224	1.852	-0.024	0.852	0.064
Emotional Reactions to Parent Leaving (M)	0.109	0.325	0.335	-0.529	0.747	0.737
SDQ Emotional Symptoms (W)	0.138	0.268	0.515	-0.387	0.663	0.606
X * W	-0.125	0.066	-1.905	-0.254	0.004	0.057
X * M	0.040	0.098	0.414	-0.151	0.232	0.679
Child Gender	-0.113	0.506	-0.223	-1.105	0.879	0.823
Child Age	-0.233	0.147	-1.582	-0.521	0.056	0.114
Jailed Parent Race	1.090	0.524	2.080	0.063	2.116	0.038

Model Summary $\chi^2(8) = 10.812$, $p = 0.213$
$R^2_{McFadden} = 0.104$, $R^2_{Cox\text{-}Snell} = 0.133$, $R^2_{Nagelkirk} = 0.178$

Child Language Delay

Variables	*B*	*S.E.*	z	95% CI LL	95% CI UL	*p*
Constant	2.216	1.288	1.721	-0.307	4.739	0.085
Distress (X)	0.224	0.222	1.012	-0.210	0.658	0.311
Emotional Reactions to Parent Leaving (M)	0.278	0.334	0.831	-0.377	0.932	0.406
SDQ Emotional Symptoms (W)	-0.056	0.290	-0.195	-0.624	0.511	0.846
X * W	-0.105	0.069	-1.514	-0.240	0.031	0.130
X * M	0.028	0.109	0.260	-0.185	0.242	0.795
Child Gender	-1.737	0.586	-2.964	-2.886	-0.588	0.003
Child Age	-0.449	0.172	-2.607	-0.787	-0.111	0.009
Jailed Parent Race	0.406	0.555	0.733	-0.681	1.494	0.464

Model Summary $\chi^2(8) = 19.571$, $p = 0.012$
$R^2_{McFadden} = 0.189$, $R^2_{Cox\text{-}Snell} = 0.227$, $R^2_{Nagelkirk} = 0.305$

Child Social Adaptive Delay

Variables	*B*	*S.E.*	z	95% CI LL	95% CI UL	*p*
Constant	2.994	1.375	2.177	0.299	5.690	0.029
Distress (X)	0.211	0.239	0.880	-0.259	0.680	0.379
Emotional Reactions to Parent Leaving (M)	-0.326	0.389	-0.838	-1.090	0.437	0.402
SDQ Emotional Symptoms (W)	-0.002	0.321	-0.006	-0.630	0.630	0.996
X * W	0.009	0.076	.0114	-0.140	0.157	0.909
X * M	-0.017	0.134	-0.128	-0.279	0.245	0.898
Child Gender	-0.872	0.603	-1.446	-2.053	0.310	0.148
Child Age	-0.590	0.190	-3.097	-0.963	-0.217	0.002
Jailed Parent Race	-0.162	0.575	-0.283	-1.289	0.964	0.778

Model Summary $\chi^2(8) = 17.611$, $p = 0.024$
$R^2_{McFadden} = 0.189$, $R^2_{Cox\text{-}Snell} = 0.207$, $R^2_{Nagelkirk} = 0.293$

Child Motor Delay

Variables	*B*	*S.E.*	z	95% CI LL	95% CI UL	*p*
Constant	1.763	1.282	1.376	-0.749	4.275	0.169
Distress (X)	0.514	0.249	2.067	0.027	1.001	0.039
Emotional Reactions to Parent Leaving (M)	0.064	0.377	0.171	-0.674	0.803	0.865
SDQ Emotional Symptoms (W)	0.767	0.327	2.348	0.127	1.408	0.019
X * W	-0.108	0.072	-1.494	-0.249	0.034	0.135
X * M	-0.103	0.114	-0.909	-0.326	0.120	0.363
Child Gender	-0.006	0.565	-0.010	-1.112	1.101	0.992
Child Age	-0.708	0.192	-3.688	-1.084	-0.332	0.000
Jailed Parent Race	0.038	0.567	0.067	-1.074	1.150	0.947

Model Summary $\chi^2(8) = 25.736$, $p = 0.001$
$R^2_{McFadden} = 0.246$, $R^2_{Cox\text{-}Snell} = 0.287$, $R^2_{Nagelkirk} = 0.384$

Note. *CI* = Confidence Interval; *LL* = Lower Level; *UL* = Upper Level; *SDQ* = Strengths and Difficulties Questionnaire

Supplementary Table 3 (Compare to Table 4). *Multiple Regression Results from PROCESS Models: Child Health.*

a. Predictors of Young Children's Health at Time 1 (*N*=76).

				95% CI		
Predictor	***β***	*SE*	*t*	LL	UL	*p*
Constant	4.478	0.399	11.216	3.681	5.275	0.000
Distress (X)	0.083	0.074	1.121	-0.065	0.231	0.266
Emotional Reactions to Parent Leaving (M)	0.114	0.107	1.066	-0.100	0.328	0.290
SDQ Emotional Symptoms (W)	-0.015	0.090	-0.171	-0.195	0.165	0.875
X * W	-0.050	0.022	-2.227	-0.094	-0.005	0.029
M * M	0.003	0.033	0.101	-0.063	0.070	0.920
Child Gender	-0.011	0.178	-0.060	-0.366	0.344	0.952
Child Age	-0.068	0.050	-1.355	-0.169	0.032	0.180
Jailed Parent Race	0.116	0.181	0.641	-0.246	0.478	0.524
Model Summary $R^2 = 0.175$, $F(8,67) = 1.774$, $p = 0.098$						

b. Predictors of Young Children's Health at Time 2 (*N*=76).

				95% CI		
Predictor	***β***	*SE*	*t*	LL	UL	*p*
Constant	4.919	0.434	11.324	4.052	5.786	0.000
Distress (X)	-0.089	0.081	-1.102	-0.250	0.072	0.275
Emotional Reactions to Parents Leaving (M)	0.064	0.117	0.552	-0.168	0.297	0.582
SDQ Emotional Symptoms (W)	-0.064	0.098	-0.651	-0.260	0.132	0.517
X * W	0.010	0.024	0.399	-0.039	0.058	0.691
X * M	-0.020	0.036	-0.554	-0.093	0.052	0.581
Child Gender	0.010	0.194	0.053	-0.376	0.397	0.958
Child Age	-0.105	0.055	-1.923	-0.215	0.004	0.059
Jailed Parent Race	-0.046	0.197	-0.233	-0.440	0.348	0.816
Model Summary $R^2 = 0.151$, $F(8,67) = 1.493$, $p = 0.176$						

Note. *CI* = Confidence Interval; *LL* = Lower Level; *UL* = Upper Level; *SDQ* = Strengths and Difficulties Questionnaire

Supplementary Table 4 (Compare to Table 2). *Multiple Regression Results from PROCESS Models: Emotional Reactions to Separation and Total Developmental Delays.*

a. *Predictors of Young Children's Emotional Reactions to Parents Leaving for Jail, Time 1 (N=76).*

				95% CI		
Predictor	***β***	*SE*	*t*	LL	UL	*p*
Constant	1.866	0.624	2.993	0.622	3.111	0.004
Witness Arrest (X)	-0.156	0.107	-1.460	-0.369	0.057	0.149
SDQ Emotional Symptoms (W)	0.168	0.106	1.591	-0.043	0.379	0.116
X * W	0.062	0.030	2.056	0.002	0.122	0.044
Child Gender	0.268	0.314	0.851	-0.360	0.895	0.398
Child Age	-0.006	0.088	-0.069	-0.182	0.170	0.945
Jailed Parent Race	-0.476	0.314	-0.517	-1.102	0.150	0.134
Witness Crime	-0.012	0.070	-0.167	-0.152	0.128	0.868
Model Summary $R^2 = 0.246$, $F(7,68) = 3.162$, $p = 0.006$						

b. *Predictors of Young Children's Emotional Reactions to Parents Leaving for Jail, Time 2 (N=76).*

				95% CI		
Predictor	***β***	*SE*	*t*	LL	UL	*p*
Constant	1.004	0.824	1.218	-0.640	2.647	0.227
Witness Arrest (X)	0.056	0.141	0.397	-0.225	0.337	0.692
SDQ Emotional Symptoms (W)	0.392	0.140	2.812	0.114	0.671	0.006
X * W	-0.022	0.040	-0.541	-0.101	0.058	0.590
Child Gender	0.011	0.415	0.026	-0.818	0.839	0.979
Child Age	0.009	0.117	0.074	-0.224	0.241	0.941
Jailed Parent Race	0.095	0.414	0.229	-0.732	0.922	0.819
Witness Crime	0.044	0.093	0.474	-0.141	0.229	0.637
Model Summary $R^2 = 0.146$, $F(7,68) = 1.662$, $p = 0.133$						

c. *Predictors of Young Children's Total Developmental Delays* (*N*=76).

				95% CI		
Predictor	***β***	*SE*	*t*	LL	UL	*p*
Constant	3.902	0.801	4.869	2.302	5.505	0.000
Witness Arrest (X)	0.266	0.124	2.148	0.019	0.514	0.035
Emotional Reactions to Parents Leaving (M)	-0.035	0.214	-0.164	-0.462	0.392	0.870
SDQ Emotional Symptoms (W)	0.193	0.181	1.062	-0.169	0.554	0.292
X * W	-0.074	0.037	-2.016	-0.147	-0.001	0.048
M * W	0.016	0.067	0.232	-0.118	0.149	0.817
Child Gender	-0.666	0.362	-1.842	-1.388	0.056	0.070
Child Age	-0.448	0.101	-4.453	-0.649	-0.247	0.000
Jailed Parent Race	0.346	0.365	0.946	-0.384	1.075	0.347

Witness Crime	0.090	0.080	1.123	-0.070	0.251	0.265
Model Summary $R^2 = 0.311$, $F(9,66) = 3.307$, $p = 0.002$						

Note. *CI* = Confidence Interval; *LL* = Lower Level; *UL* = Upper Level; *SDQ* = Strengths and Difficulties Questionnaire

Supplementary Table 5 (Compare to Table 3). *Logistic Regression Results from PROCESS Models: Specific Developmental Delays.*

	**Child Academic Delay**						**Child Language Delay**					
				95% CI						95% CI		
Variables	*B*	*S.E.*	z	LL	UL	*p*	*B*	*S.E.*	z	LL	UL	*p*
Constant	-0.470	1.173	-0.401	-2.769	1.829	0.688	2.227	1.296	1.719	-0.313	4.767	0.086
Witness Arrest (X)	0.390	0.193	2.024	0.012	0.767	0.043	0.166	0.185	0.897	-0.197	0.529	0.370
Emotional Reactions to Parent Leaving (M)	0.114	0.331	0.345	-0.534	0.762	0.730	0.268	0.335	0.798	-0.390	0.925	0.425
SDQ Emotional Symptoms (W)	0.156	0.272	0.572	-0.378	0.689	0.567	-0.072	0.297	-0.242	-0.654	0.510	0.809
X * W	-0.114	0.056	-2.051	-0.223	-0.005	0.040	-0.085	0.057	-1.500	-0.197	0.026	0.134
X * M	0.041	0.099	0.416	-0.153	0.236	0.677	0.034	0.111	0.306	-0.184	0.253	0.759
Child Gender	-0.107	0.516	-0.207	-1.119	0.905	0.836	-1.772	0.610	-2.906	-2.968	-0.577	0.004
Child Age	-0.237	0.148	-1.598	-0.528	0.054	0.110	-0.448	0.173	-2.589	-0.787	-0.109	0.010
Jailed Parent Race	1.130	0.535	2.111	0.081	2.180	0.035	0.435	0.559	0.778	-0.661	1.530	0.437
Witness Crime	-0.014	0.116	-0.120	-0.240	0.213	0.904	0.029	0.127	0.224	-0.221	0.278	0.823
	Model Summary $\chi^2(9) = 11.708$, $p = 0.230$ $R^2_{McFadden} = 0.113$, $R^2_{Cox\text{-}Snell} = 0.143$, $R^2_{Nagelkirk} = 0.191$						Model Summary $\chi^2(9) = 19.660$, $p = 0.020$ $R^2_{McFadden} = 0.190$, $R^2_{Cox\text{-}Snell} = 0.228$, $R^2_{Nagelkirk} = 0.306$					

	**Child Social Adaptive Delay**						**Child Motor Delay**					
				95% CI						95% CI		
Variables	*B*	*S.E.*	z	LL	UL	*p*	*B*	*S.E.*	z	LL	UL	*p*
Constant	3.342	1.512	2.211	0.379	6.305	0.027	1.771	1.278	1.385	-0.734	4.276	0.166
Witness Arrest (X)	0.086	0.198	0.433	-0.302	0.473	0.665	0.387	0.207	1.868	-0.019	0.793	0.062
Emotional Reactions to Parent Leaving (M)	-0.486	0.382	-1.270	-1.235	0.264	0.204	0.046	0.375	0.112	-0.693	0.776	0.911
SDQ Emotional Symptoms (W)	-0.202	0.362	-0.558	-0.911	0.507	0.577	0.750	0.327	2.295	0.109	1.390	0.022
X * W	0.016	0.062	0.252	-0.106	0.137	0.801	-0.087	0.059	-1.463	-0.203	0.029	0.144
X * M	0.055	0.138	0.398	-0.215	0.325	0.691	-0.096	0.113	-0.844	-0.318	0.127	0.399
Child Gender	-1.246	0.667	-1.868	-2.553	0.061	0.062	-0.029	0.571	-0.051	-1.148	1.090	0.959
Child Age	-0.668	0.221	-3.023	-1.100	-0.235	0.003	-0.695	0.190	-3.656	-1.068	-0.322	0.000
Jailed Parent Race	0.058	0.611	0.094	-1.140	1.255	0.925	0.074	0.569	0.131	-1.041	1.189	0.896
Witness Crime	0.338	0.143	2.357	0.057	0.619	0.018	0.018	0.127	0.138	-0.231	0.266	0.890
	Model Summary $\chi^2(9) = 23.757$, $p = 0.005$ $R^2_{McFadden} = 0.255$, $R^2_{Cox\text{-}Snell} = 0.268$, $R^2_{Nagelkirk} = 0.380$						Model Summary $\chi^2(9) = 25.073$, $p = 0.003$ $R^2_{McFadden} = 0.240$, $R^2_{Cox\text{-}Snell} = 0.281$, $R^2_{Nagelkirk} = 0.376$					

Note. *CI* = Confidence Interval; *LL* = Lower Level; *UL* = Upper Level; *SDQ* = Strengths and Difficulties Questionnaire

Supplementary Table 6 (Compare to Table 4). *Multiple Regression Results from PROCESS Models: Child Health.*

a. *Predictors of Young Children's Health at Time 1 (N=76).*

				95% CI		
Predictor	***β***	*SE*	*t*	LL	UL	*p*
Constant	4.481	0.404	11.087	3.674	5.288	0.000
Witness Arrest (X)	0.079	0.063	1.261	-0.046	0.204	0.212
Emotional Reactions to Parent Leaving (M)	0.120	0.108	1.107	-0.096	0.335	0.272
SDQ Emotional Symptoms (W)	-0.010	0.091	-0.106	-0.192	0.173	0.916
X * W	-0.042	0.019	-2.266	-0.079	-0.005	0.027
M * M	0.002	0.034	0.045	-0.066	0.069	0.964
Child Gender	0.004	0.182	0.022	-0.360	0.368	0.983
Child Age	-0.070	0.051	-1.382	-0.171	0.031	0.172
Jailed Parent Race	0.112	0.184	0.609	-0.256	0.480	0.545
Witness Crime	-0.017	0.041	-0.413	-0.098	0.064	0.681
Model Summary $R^2 = 0.176$, $F(9,66) = 1.562$, $p = 0.145$						

b. *Predictors of Young Children's Health at Time 2 (N=76).*

				95% CI		
Predictor	***β***	*SE*	*t*	LL	UL	*p*
Constant	4.853	0.429	11.321	3.997	5.709	0.000
Witness Arrest (X)	-0.071	0.070	-1.024	-0.210	0.068	0.310
Emotional Reactions to Parents Leaving Time 2 (M)	0.139	0.110	1.264	-0.081	0.358	0.211
SDQ Emotional Symptoms (W)	-0.073	0.081	-0.895	-0.235	0.089	0.374
X * W	0.009	0.019	0.471	-0.030	0.048	0.640
X * M	-0.027	0.028	-0.951	-0.084	0.030	0.345
Child Gender	0.047	0.194	0.241	-0.341	0.434	0.811
Child Age	-0.103	0.055	-1.863	-0.212	0.007	0.067
Jailed Parent Race	-0.050	0.195	-0.256	-0.440	0.340	0.799
Witness Crime	-0.038	0.044	-0.862	-0.127	0.050	0.392
Model Summary $R^2 = 0.176$, $F(9,66) = 1.567$, $p < 0.144$						

Note. *CI* = Confidence Interval; *LL* = Lower Level; *UL* = Upper Level; *SDQ* = Strengths and Difficulties Questionnaire
